# Age and gender differences in objective sleep properties using large-scale body acceleration data in a Japanese population

**DOI:** 10.1038/s41598-021-89341-x

**Published:** 2021-05-11

**Authors:** Li Li, Toru Nakamura, Junichiro Hayano, Yoshiharu Yamamoto

**Affiliations:** 1 Graduate School of Engineering Science, Osaka University, 1-3 Machikaneyama, Toyonaka, Osaka 560-8531 Japan; 2 Intasect Communications, Inc., 3-1 Ogawa-cho, Kanda, Chiyoda-ku, Tokyo, 101-0052 Japan; 3 Graduate School of Medical Sciences, Nagoya City University, 1 Kawasumi, Mizuho-cho, Mizuho-ku, Nagoya, 467-8601 Japan; 4 Graduate School of Education, The University of Tokyo, 7-3-1 Hongo, Bunkyo-ku, Tokyo, 113-0033 Japan

**Keywords:** Ageing, Sleep, Biomedical engineering

## Abstract

Using large-scale objective sleep data derived from body acceleration signals of 68,604 Japanese residents ranging from adolescents to the elderly (10–89 years old), we found significant age- and gender-related differences in sleep properties (timing, duration, and quality) in real-life settings. Time-in-bed and total sleep time (TST) showed a U-shaped association with age, indicating their decrease in adulthood following their increase in the elderly. There was a remarkable shift in sleep phase toward earlier bedtime and earlier wake time with increasing age (> 20 years), together with worsening of sleep quality, which is estimated by sleep efficiency (SE) and wake time after sleep onset. Gender comparisons showed that TST was shorter in women than in similarly aged men, which is much evident after the age of 30 years. This was associated with later bedtimes and greater age-related deterioration of sleep quality in women. Compared to men in the same age group, women over age 50 demonstrated a greater reduction in SE with aging, due mainly to increasing durations of nighttime awakening. These differences can be attributed to several intricately intertwined causes, including biological aging as well as socio-cultural and socio-familial factors in Japan. In conclusion, our findings provide valuable insights on the characteristics of Japanese sleep habits.

## Introduction

Sleep properties, such as timing and quality of sleep, significantly differ according to age and gender. Investigating alterations of sleep properties related to these factors is important to understand the human normative sleep or pathological sleep conditions. Most previous epidemiological studies with questionnaires^1,2^, objective measures (e.g., actigraphy)^2–4^, and meta-analysis^5,6^, have consistently shown that sleep durations tend to decrease from childhood to adulthood, along with worsening of sleep quality, such as reductions in total sleep time (TST) and sleep efficiency (SE), as well as an increase in the amount of time spent awake after sleep onset. Furthermore, the higher prevalence of sleep problems and longer sleep in women than men have been reported in a most epidemiological surveys using population of Western countries^4,7–11^.

A few epidemiological studies have provided the subjective sleep profiles of Japanese populations^12–16^, although there was no survey primarily focusing on age and gender effects of sleep. One study examining the mortality risk of sleep duration in adults aged 40–79 years reported a longer sleep time in men than women, along with an increasing tendency of sleep duration with age^16^. Similar results were also reported in other surveys of insomnia, although they reported opposite results in adults < 40 years^13,15^. Sleep quality estimated by self-reported complaints was also influenced by age and gender differences in a Japanese population. Various surveys consistently reported both the higher frequency of sleep complaints in female and an increase of the prevalence of insomnia with age^12,15^, as reported in most population-based studies performed in other countries^4,5,7–9,17^. Nevertheless, those surveys provided an important knowledge on sleep profiles of Japanese population. They all relied on subjective assessment and lacked the objective aspects. To the best of our knowledge, a population-based sleep study focusing on age and gender effects using objective sleep measures has never been conducted.

Recently, we gathered large-scale 24-h body acceleration data recorded from > 80,000 individuals residing in Japan, including those recorded during sleep in real-life settings^18–21^. Since recordings were continued even during sleep, an analysis of the database provided an opportunity to conduct sleep studies using objective measures based on body acceleration (i.e., actigraphy) in a Japanese population. Therefore, in our previous study^22^, we conducted the sleep–wake annotations of each acceleration data stored in the database in order to derive the objective sleep properties and we then validated our annotations. In this study, using the objective sleep data annotated in our previous study, we examined age- and gender-differences in sleep properties (timing and quality of sleep), including timing of go to bed/wake-up and sleep quality (e.g., sleep efficiency, or wake time after sleep onset).

## Materials and methods

### ALLSTAR database

We used a database of 24-h electrocardiography (ECG) and tri-axial acceleration data constructed by the ALLSTAR research project^18–21^ to examine age- and gender-related variations in parameters of habitual sleep in a Japanese population. The database of the ALLSTAR research project has been explained in detail elsewhere^18–21^. Briefly, the project was started in 2010, with the cooperation of Suzuken Co., Ltd (Nagoya, Japan), the owner of the data, and researchers from seven universities across Japan (including J.H. and Y.Y.), to establish methods of evaluating the health impacts of environmental factors using Holter ECG recording data gathered from all over Japan. The project seeks to enhance the utility of Holter ECG and bodily acceleration data in healthcare fields and to facilitate predictive and preventive healthcare to increase longevity.

The database comprises 24-h ambulatory Holter recording data recorded with series of Cardy device (Cardy 2, Cardy 2P, Cardy 203, Cardy 301, Cardy 302 Mini and Max, Cardy 303 pico, and Cardy 303 pico+; Suzuken Co., Ltd., Japan) by medical the facilities for various clinical purposes, including screening and diagnosis of diseases and evaluation of treatment effects. The data recorded by the medical facilities in Japan were sent to one of the Suzuken’s ECG analysis centers located in Sapporo, Nagoya, and Tokyo, in Japan. All data were anonymized and stored in the database, except cases where subjects disagree on the use of their data for the project. The database also stored each subject’s age and gender, as well as each recording’s date, time, and location (as defined by the postal code of each medical facility). The ECG data were analyzed with Holter ECG analyzers (Cardy Analyzer05, Suzuken Co., Ltd., Nagoya, Japan) by skilled medical technologists; temporal positions of all R-waves were detected, all QRS complexes were annotated, and all errors in automated analysis were corrected manually by skilled clinical technologists.

After the release of new Holter recorders with tri-axial accelerometers (Cardy 303 pico and Cardy 303 pico+; Suzuken Co., Ltd., Japan), 24-h body acceleration signals were recorded along with the ECG data. The accelerometer (Tri-axial piezoresistive accelerometer: HAAM-326B, HOKURIKU electric industry Co., Ltd.,) sensed up to ± 3*g* (m/s^2^) (*g*: gravitational acceleration) in medio-lateral, antero-posterior, and vertical directions. Acceleration data were digitized at 31.25 Hz with 10-bit resolution by an analog-to-digital converter.

Holter monitoring is one of the ambulatory assessments with which recordings are conducted in natural daily circumstances and not in laboratory settings, without any restrictions on subjects’ daily activities. Patients are instructed to go about their days while they are monitored and are also wear a device before sleeping. Thus, the data of ALLSTAR database included continuous ECG and acceleration data during sleep, which allows us to evaluate sleep properties in real-life settings. The old-type Holter recorders were not water-resistant; therefore, participants wearing such devices were instructed to refrain from showering or bathing during the monitoring.

### Samples

**Figure 1:**
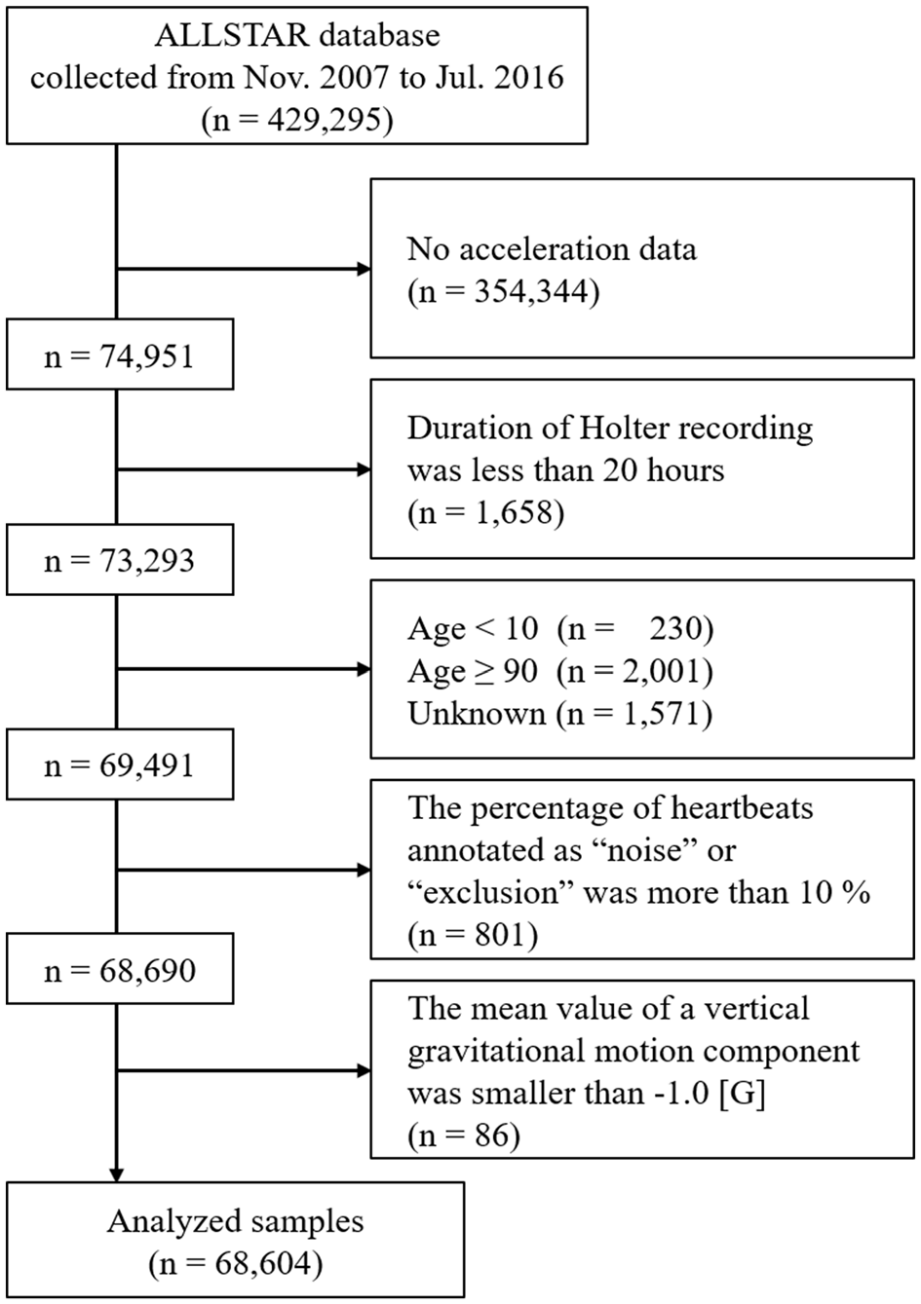
Flow chart of data availability.

We used the tri-axial acceleration data stored in the database (74,951 subjects: Fig. 1). From the database, we extracted subjects who met the following criteria: (1) Holter recording duration longer than 20 h, (2) age at recording between 10 and 89 years, (3) percentage of heartbeats annotated as “noise” or “exclusion” less than 10%, and (4) mean value of the vertical gravitational motion component (GMC)^23,24^ over the entire recording period larger than − 1.0 g [m/s^2^]. As explained below (also see Supplementary Information), each GMC signal approximates the Earth’s gravitational acceleration applied to an accelerometer in each direction. Therefore, GMCs are highly correlated with upper-body postures; for example, values of the vertical GMC were close to − 1.0 when subjects were in an upright position (e.g., standing or sitting), and zero when they were in a horizontal position (e.g., lying down). The fourth criterion usually could not be satisfied when Holter recordings were not properly conducted due to device malfunction or incorrect usage.

Overall, acceleration data from 68,604 individuals (30,485 men, 37,951 women, and 168 individuals with unknown gender; mean age: 49.7 ± 2.8 years) fulfilled all inclusion criteria (Fig. 1). These were recorded by more than 1500 medical facilities in 47 prefectures in Japan. In the successive analysis, we categorized the individuals into eight age groups defined by 10-year intervals. The distribution of age groups is shown in Table 1. This study was approved by the ethics committee of Osaka University.

**Table 1:** Age and gender distributions of Holter acceleration data.

Age group	Age range (years old)	Sample number	Male/female (gender unknown)
10s	10–19	1314	684/625 (5)
20s	20–29	1421	655/761 (5)
30s	30–39	2816	1205/1602 (9)
40s	40–49	5448	2444/2990 (14)
50s	50–59	7361	3536/3804 (21)
60s	60–69	14,728	6956/7728 (44)
70s	70–79	21,710	9517/12,156 (37)
80s	80–89	13,806	5488/8285 (33)
Total		68,604	30,485/37,951 (168)

### Sleep–wake annotations by machine learning

Minute-by-minute sleep–wake annotations were conducted using an acceleration-based sleep–wake classifier that was developed and validated by the authors in a previous study^22^. The details are described in the Supplementary Information. Here, we briefly introduce our sleep–wake annotation method.

A support vector machine (SVM)^25,26^ was employed to construct a sleep–wake classifier. The classifier converted the tri-axial trunk acceleration data measured by a Holter recorder into a sequence of “sleep” and “wake” labels with 1-min time resolution, using statistical features extracted from acceleration data. The features, an input vector to the SVM-based classifier, were calculated and selected as follows: acceleration data in each direction were separated into a bodily motion component (BMC) and a gravitational component (GC)^23,24^ by a low-pass filter. The BMC is related to the amount of physical activity and the GC reflects upper-body postures. Each component was divided into a series of 1-min windows. For each window, basic statistics, signal magnitude, upper-body tilt angles and local variances of physical activity (*TPower*; refer to Supplementary Information) were calculated from the GC and BMC signals, respectively. Factor analysis was applied to classify these features into subgroups of highly-correlated features. Trunk angles and *TPower* were selected as representative features from resultant subgroups. We then constructed an SVM-based classifier using these features and validated the performance in minute-by-minute sleep–wake classification.

In training and testing of the classifiers, we used the minute-by-minute sleep–wake estimates derived from wrist activity data that were simultaneously measured during a Holter recording using an actigraph (Ambulatory Monitoring Inc., Ardsley, NY, USA) as ground truth data, since the polysomnography (PSG) data were not available in this study. The AMI actigraph has been shown to correctly distinguish sleep from wakefulness with high accuracy (> 90%)^27,28^ and high sensitivity (> 95%)^28,29^ compared to PSG, the gold standard for sleep assessment. Therefore, the AMI actigraph has been widely used in sleep studies, especially in real-life settings, as a substitute for PSG^27,30,31^.

Our previous work reported that the comparison of sleep–wake estimates between our SVM-based classifier and AMI actigraph showed good agreement (accuracy = 94.4% ± 3.8%, specificity = 94.2% ± 5.2%, sensitivity = 94.8% ± 3.9%, and F1-score = 92.0 ± 4.5)^22^. This indicates that our algorithm had comparable performance in sleep–wake classification with the AMI actigraph (Supplementary Information).

### Sleep parameters

We examined seven sleep parameters^30,32^: in-bed time (IBT), get-up time (GUT), time-in-bed (TIB), sleep latency (SL), wake time after sleep onset (WASO), total sleep time (TST), and sleep efficiency (SE). In-bed time is the clock time when a subject got in bed to sleep and then switched the light off. Get-up time is the clock time when a subject finally awakened in the morning. In an actigraphic study, IBT and GUT are often determined using data from the event marker of an actigraph, sleep diary, or an ambient light senor^5^. However, since such data were not available in the database, we determined those timings using trunk tilt angles and a local variance of acceleration data (*TPower*). The detailed algorithm and its validation are described in Supplementary Information. TIB is the duration that the subject spent in bed. Technically, it can be derived by subtracting the time a subject went to bed from the time that subject arose. In this study, it is defined as the time between IBT and GUT. SL refers to the time it took a subject to fall asleep. It is defined as the number of minutes between IBT and sleep onset, where sleep onset is the time at the start of the first 10 consecutive minutes of sleep after IBT. WASO is the total minutes a subject was awake from sleep onset to GUT. TST refers to the number of minutes a subject was asleep between sleep onset and GUT. It can be calculated by subtracting SL and WASO from TIB. SE is the ratio of TST to TIB multiplied by 100, or [(TIB − SL − WASO)/TIB] × 100. The validation of sleep parameters (TIB, TST, SL, SE, and WASO) scored by our method was conducted by comparing them with those derived by the AMI actigraph, showing good agreement with both scoring (Supplementary Information)^22^.

### Statistics

Normality of data was checked by Kolmogorov–Smirnov test, Cramter–von Mises test, and Anderson–Darling, together with a visual inspection of quantile–quantile plots (q–q plots). The q–q plots indicated large deviations from a normal distribution in all sleep parameters, especially SE, SL, and WASO (Fig. S5). In addition, all tests indicated a significant (*p* < 0.01) divergence from normality.

We employed a generalized linear model (GLM) to test age and gender effects on each sleep parameter. In GLM analysis, residuals do not necessarily need to follow a Gaussian distribution, but are allowed to take an exponential family of probability distributions. In addition, GLM has the link function, which provides the transformation of the expected values of outcome, which is capable of representing a non-linear association between predictors and outcomes. Considering the non-normality of sleep parameters, we assumed both a normal and gamma distribution for residuals. Furthermore, we tested the following four functions for link function: identity, log, inverse, and inverse squared. Therefore, we considered all possible pairs of a distribution and a link function and selected the best model for the sleep parameters based on Akaike information criteria.

The sleep parameter values were assumed to follow a gamma distribution. The month during which Holter recording was performed was also incorporated as an independent categorical variable to account for possible seasonal variations. In addition to the main effects of the categorical variables (age, gender, and month), the interaction term between age and gender was considered as a potential factor affecting sleep parameter values. When the interaction term was not significant, a separate GLM that excluded it from the model was created, and the analysis was performed again. If the interaction term was significant, we stratified the data by age or gender and then tested simple main effects (i.e., pairwise comparisons) with the Bonferroni correction for multiple comparisons.

In a GLM regression, we used the Bonferroni correction to adjust *p*-values derived from models. In this study, we compared the results of the 20s age group with those of the other age groups to examine age-related differences in sleep parameters. All statistical tests were conducted using SAS software version 9.04 (SAS Institute, Cary, NC, USA). A conservative value of *p* < 0.01 was considered statistically significant to avoid potential inferential biases caused by a large sample size^33,34^. All data are expressed as the mean ± standard error of the mean.

## Results

The GLM analysis showed significant main effects of age and gender on all sleep parameters. Note that the pair of normal distribution and identity function was selected for TST, in-bed-time, get-up time, and SE, while the logarithmic link function was selected for TIB. Combination of a gamma distribution and the logarithmic link function was the best for SL and WASO. The interaction terms were also significant, except for get-up time. Results of all statistical tests are summarized in Tables S2–S3.

### Age effects on sleep parameters

**Figure 2:**
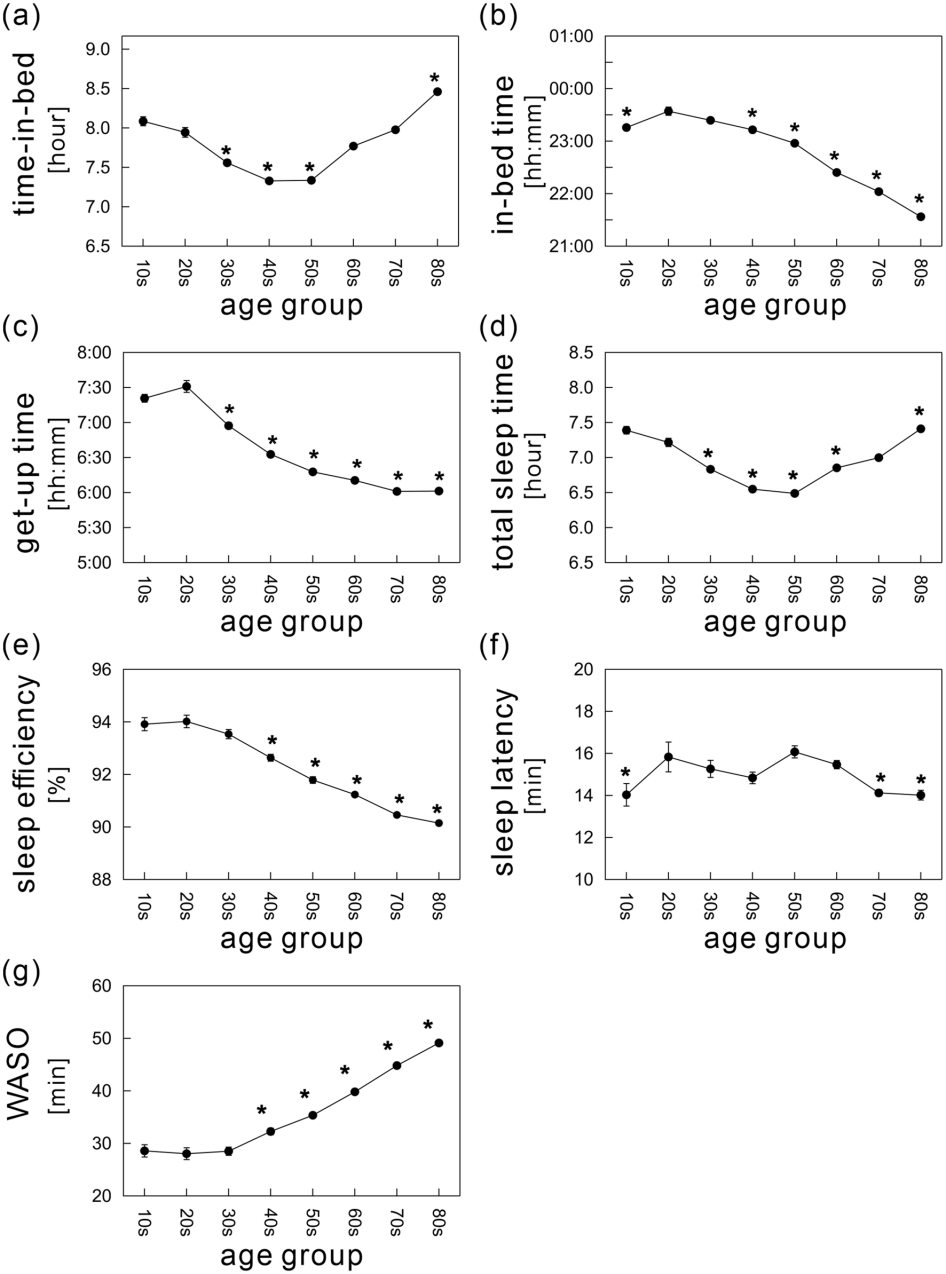
Age-dependent differences in sleep parameters: (**a**) time-in-bed (TIB), (**b**) in-bed time, (**c**) get-up time, (**d**) total sleep time, (**e**) sleep efficiency, (**f**) sleep latency, and (**g**) wake time after sleep onset (WASO). The mean values of each sleep parameter are shown as a function of age, ranging from age 10 to 89 years divided into eight age groups with 10-year intervals (solid black circles). The error bars indicate the standard error of the mean. *Indicates significance relative to the 20s age group (*p* < 0.01).

Figure 2 shows the age-related differences in each sleep parameter (detailed values are shown in Table S2). Note that sleep parameter values plotted as a function of continuous variable of age were also provided (Fig. S6).

There was a U-shaped association between age and TIB, with TIB ranging from 7.3 to 8.5 h and the nadir of the curve in the 40s age group (Fig. 2a). This pattern was also confirmed when age was treated as continuous variable (Fig. S6). When compared with the 20s age group, TIB durations showed significant decreases in middle-aged adults (i.e., the 30s, 40s, and 50s age groups), and significant increases in younger and older individuals (the 10s and 80s age groups, respectively). The mean IBT was latest in the 20s age group (11:34 p.m.), and then advanced linearly after age 20 by 20.1 min per decade of age (Fig. 2b). The earliest IBT was 9:34 p.m., in the 80s age group. The mean GUT was also latest in the 20s age group (7:31 a.m.), and advanced exponentially with age (Fig. 2c); the rate of decline was steepest between the 20s and 40s age groups, and then gradually stabilized. The mean GUT of subjects in their 80s was 6:01 ± 0:01 a.m. Wake time shifted earlier by a mean of 14.9 min per decade of age after age 20. Both mean IBT and GUT showed an increasing tendency from the age group of 10–20s. These were visually much clearer in the figures plotted as a function of continuous variable of age between 0 and 20 years (Fig. S6c,d).

The mean TST also showed a U-shaped association with age (Fig. 2d); TST ranged from 6.5 to 7.4 h, with the shorter duration in in the working population (the 30s–60s age groups).

The mean SE declined linearly after 20 years of age (Fig. 2e). The decrease was particularly evident after 40 years of age. This finding included significant gender differences in SE, as noted below.

Although we found a significant main effect of age on SL, the mean SL ranged from 14.0 to 16.1 min and the differences among age groups were relatively small (Fig. 2f). These subtle variations indicate that the age effect was not physiologically relevant.

The mean WASO over age 30 years showed a striking and monotonic increase of 4.1 min per decade, with no noticeable change found in subjects under age 30 years (Fig. 2g). By definition, a decrease in SE is associated with both a longer SL and a longer WASO. Considering the subtle differences in SL, the worsening SE was mainly attributed to increasing awake time during the night. Gender comparisons below further probe the significant contribution of data from female subjects to the striking increase in WASO with aging.

### Gender effects on sleep parameters

**Figure 3:**
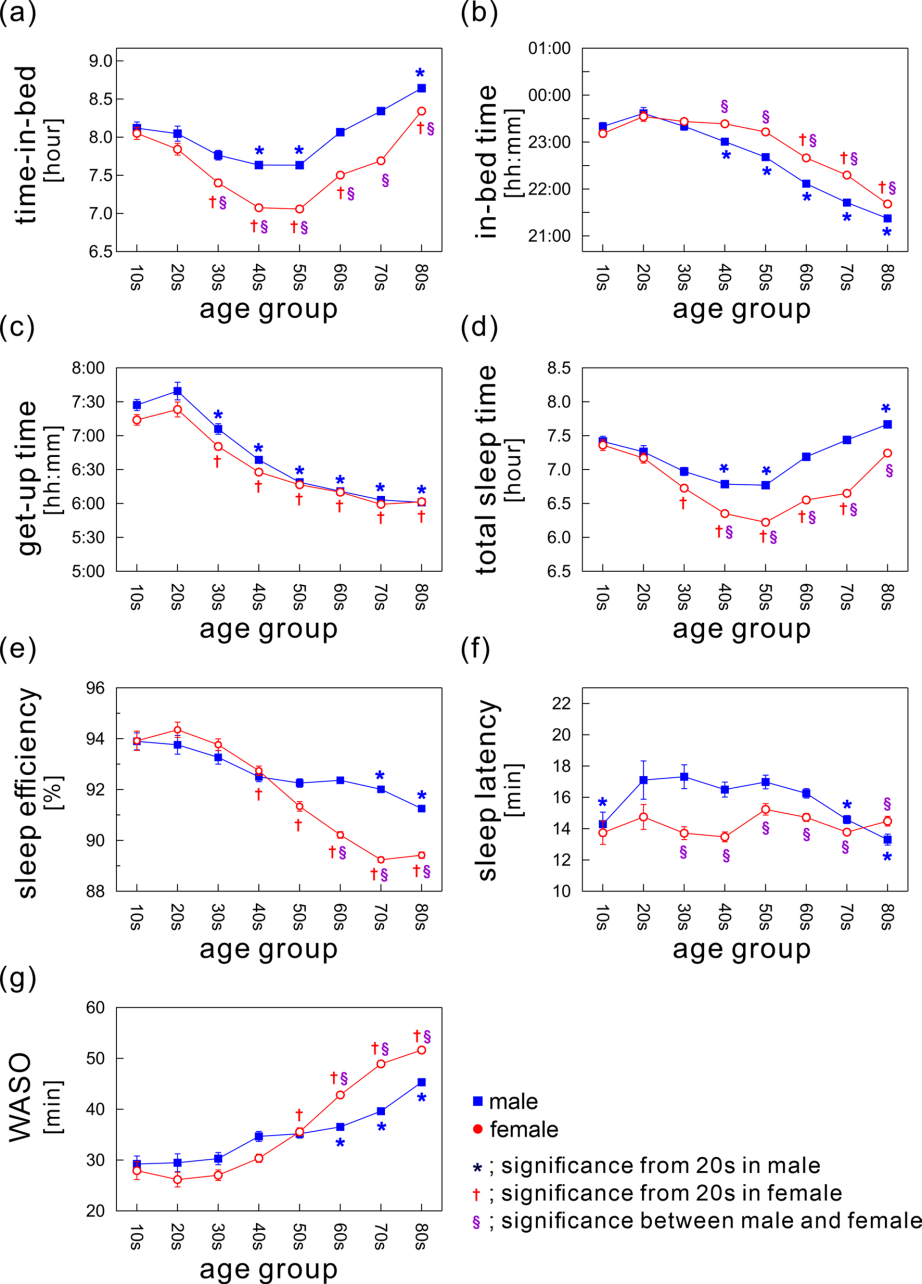
Gender differences in sleep parameters: (**a**) time-in-bed (TIB), (**b**) in-bed time, (**c**) get-up time, (**d**) total sleep time, (**e**) sleep efficiency, (**f**) sleep latency, and (**g**) wake time after sleep onset (WASO). The mean values of each sleep parameter for each gender are shown as a function of age, ranging from age 10 to 89 years divided into eight age groups with 10-year intervals (men, blue rectangles; women, open red circles). The error bars indicate the standard error of the mean. * and ^†^ Indicate significant age-dependent changes in males and females, respectively, compared with the 20s age group (*p* < 0.01). ^§^Indicates significant gender differences (*p* < 0.01).

Figure 3 shows gender effects on sleep parameters. The figures plotted as a function of continuous variable of age were also provided (Fig. S7). In addition, the detailed values are shown in Table S2.

The mean TIB durations in both genders showed a U-shaped pattern, and the shortest durations were in middle-aged subjects (Fig. 3a) even when the data were stratified by gender. The shortest mean TIB duration was in the 50s age group in both genders (men, 7.64 ± 0.04 h; women, 7.06 ± 0.03 h). Gender comparisons showed that over age 30 years, TIB durations in women were consistently and significantly shorter than those in men. On average, women slept 30.1 ± 0.5 min less than men. Similar results were also confirmed for TST (Fig. 3d). The mean TST in women over age 30 years was significantly shorter than that in men, with a mean gender difference of 30.7 ± 0.4 min.

An age-dependent advance in IBT was found in both genders (Fig. 3b), while women over age 40 years had significantly later IBT compared to similarly aged men; IBT advanced linearly by 19.6 min per decade of age in males and by 20.5 min per decade of age in females. In addition, the overall mean difference in IBT between genders after age 40 years was 28.3 ± 0.5 min.

The mean GUT significantly advanced in adults of both genders over age 20 years, with no significant gender differences in any age group (Fig. 3c).

The mean SE in males was almost constant across age groups (overall mean from ages 20 to 89 years, 92.5 ± 0.2%), with a significant downward trend with increasing age (Fig. 3e). By contrast, women showed a distinctive decline with age beginning in the 20s age group.

Age-related differences in mean SL were significant but subtle in both genders (range of mean SL values: 13.3 ± 17.3 min in males, 13.5 ± 15.2 min in females), while a significant main effect of age was confirmed in males (Fig. 3f). Gender comparisons showed that among subjects aged 30–79 years, men took slightly longer to fall asleep than women.

The WASO duration increased with age in adults of both genders, a trend that was more evident in women (Fig. 3g). The mean WASO duration in females increased from 26.2 ± 1.4 min to 51.7 ± 0.7 min between ages 20 and 89 years, with an increase of 3.6 min per decade of age. This striking increase in WASO duration resulted in a remarkable decline of SE in females. In males, the increase in WASO duration between ages 20 and 89 years was 2.3 min per decade of age. Gender comparisons showed that the WASO duration in subjects over age 60 years was significantly longer in women than men.

## Discussion

In this study, we investigated sleep parameters that were objectively scored from the trunk acceleration data of 68,604 residents of Japan and found significant age and gender effects on habitual sleep. Our study is the largest to describe objective sleep parameters in the Japanese population and the first to report age and gender differences in sleep properties recorded in real-life settings using objective sleep assessment.

It is widely acknowledged that sleep durations decrease with age, together with worsening of sleep quality^5,6^. However, surprisingly, we found an inconsistent result in the sleep durations, such that both TIB and TST exhibited a U-shaped pattern with increasing age between 10 and 89 years, with nadir of the curve in middle age. The consistent trends were also confirmed when data were shown as a continuous variable of age. These imply that durations are longer in both younger adults and teenagers and that a remarkable reduction is observed in the middle-age, followed by an increase in the elderly.

The decreasing tendency of sleep durations in adulthood, especially in the middle-age, have been reported in several epidemiological studies using self-reported questionnaires^12,15,35^, objective sleep measures (e.g., actigraphy)^36^, or systematic meta-analysis^5^, although comparability among studies is hampered to some extent by differences in methodologies or definitions of sleep duration. The effects of aging on sleep durations are often discussed in the context of socio-cultural factors, such as work status^36–38^, along with chronobiological aging^39–43^. Indeed, our results demonstrated the increase of sleep durations after the age of retirement (in Japan, individuals retire between 60 and 65 years of age). This would support the contribution of socio-cultural factors to shorter sleep durations, especially among middle-aged Japanese.

The increase of sleep durations in the elderly is one of the distinguishing results of this study. Although population-based research in Japan remains limited, several questionnaire-based studies support our results^12–16,44^. For instance, a survey on insomnia, which enrolled outpatients of general hospitals, reported that the elderly slept longer than younger people^12^. Other population-based studies of adults aged over 20 years also reported longer sleep durations in the elderly when compared with middle-aged adults^14,45^. Furthermore, a recent epidemiological study demonstrated prolonged sleep durations in the elderly (65–89 years old) when compared to the young (15–39 years old) and middle-aged adults (40–64 years old)^44^. Although these studies reported slightly different sleep durations for each age group, they varied in a range of about 6.5–6.8 h in the middle-age and about 7.0–7.7 h in the elderly. These ranges are comparable with our results of TST. One possible reason for the longer sleep among the elderly has to do with the Japanese cultures or health education (sleep literacy). For instance, a Japanese elderly is likely to stay in bed for a longer time, believing that longer sleep (e.g., more than 8 h) is better for his/her health. Indeed, such a behavioral pattern can be clearly seen from the increase of TIB in the elderly (Fig. 2a). We thus think that the U-shaped patterns in sleep durations would be specific to Japanese populations.

Comparing our results with sleep recommendations by American National Sleep Foundation (NSF)^6,46,47^ may provide a clearer insight (Table S5). The NSF recommendations for sufficient daily sleep durations across lifespan is as follows: 11–14 h for toddlers (1–2 years), 10–13 h for preschoolers (3–5 years), 9–11 h for school-aged children (6–13 years), and 8–10 h for teenagers (14–17 years). Seven to 9 h was recommended for adults (young adults: 18–25 years, adults: 26–40 years, and middle-aged adults: 41–65) and 7–8 h of sleep was recommended for older adults (> 65 years). In all age groups of our study population, more than 60% of the individuals were outside the recommended ranges and about 50% of the individuals slept less than recommended. Especially, the rate of less sleep was striking in younger groups (about 75% in children and 68% in teenagers). These would reflect the recent social problems of shorter sleep in Japanese, especially in children and teenagers^48^.

We found a remarkable time shift toward earlier bedtimes and earlier wake times with increasing age between 20 and 89 years, thus indicating a phase advance of sleep–wake cycles with aging. A variety of population-based studies have consistently reported a similar result using both subjective^12,35^ and objective assessments^3,4,36^, as well as cross-country meta-analysis^1^. Consistent reports of a systematic phase advance across different population groups suggest the presence of an underlying endogenous physiological mechanism; alterations in the output of the human circadian pacemaker may cause the systematic phase advance, as discussed in previous studies^42,43^.

Our results on gender differences in sleep durations were also unique. In contrast to women in other countries^1,3,4,36^, Japanese women slept 30 min shorter than similarly aged men on average. According to the questionnaire-based survey conducted by the Organization for Economic Co-operation and Development (OECD)^48^, women tend to sleep longer than men in almost all OECD countries, with just a few exceptions (Japan, India, Mexico, Estonia), and among these exceptions, the Japanese results were an extreme case. This international survey supports our finding, while it reported the shorter gender difference of 16 min than our result. Also, shorter sleep in Japanese women was reported in several studies^12–16^, although these studies adopted the subjective assessment method. The overall gender differences reported by these studies varied between 8–24 min, whereas some epidemiological studies showed that sleep durations were shorter in men until the age of 40 years^13,15^. One survey demonstrated an increasing tendency of gender difference with age: young group (20–39 years; gender difference: 1.2 min), middle group (40–59 years; 19.8 min), and elderly group (> 60 years; 23.4 min)^14^. This also supports our finding that shorter sleep in women was more evident after the age of 30 years.

One possible reason why women sleep for a shorter time than men and why the gender difference becomes much evident after the age of 30 years is gender inequality in areas, such as housework burdens or child-rearing. Most Japanese women are likely to experience a variety of major life events, such as marriage or childbirth, by their late 30s, and transitions into marriage and/or motherhood usually increase the housework burden of women rather than men^49–51^ due to traditional gender expectations^52,53^. The later bedtime that gradually becomes more evident in women after age 30 years appears to be due to increasing burdens associated with life stage transitions. Considering these, we opine that a shorter sleep in women might be characteristic to the Japanese population.

We found a progressive reduction in sleep efficiency with age, which was more evident after age 40 years and was mainly caused by increasing durations of nighttime awakening. This deterioration of sleep quality in the elderly might be explained by associations between chronobiological aging and sleep disturbances (e.g., fragmented sleep or early morning awakening), as reported in various studies^39–42^.

Furthermore, we found that such reduction of sleep quality estimated by SE and WASO was more prominent in women. As reported in several studies in different countries^4,7^, Japanese women slept with lower quality than men, especially women over the age of 50 years. In addition to the contributions of biological aging, menopause-related insomnia, persisting in postmenopausal states, might partly account for the remarkable decline of sleep quality in females. There is some evidence^54–56^ that menopausal and postmenopausal women frequently report sleep complaints, including nighttime awakening with difficulty falling back asleep, possibly linked with characteristic concomitant menopause symptoms (e.g., vasomotor symptoms such as hot flashes and night sweats, or mood swings) mediated by rapid changes in the ovarian hormonal milieu. This is apparently related with the higher prevalence of insomnia during menopause and postmenopause^56–58^.

The worsened sleep quality in women is possibly linked with gender differences in the prevalence of insomnia or psychiatric disorders (e.g., depression). Generally, the frequency of self-reported sleep problems, dissatisfaction, or complaints, is higher in women than men^17,59,60^, even in Japan^61,62^. Meanwhile, a lifetime prevalence of major depressive episodes is higher in women at puberty and is related with insomina^63–65^. According to the consortium of psychiatric epidemiology of the World Health Organization, Japanese women showed highest odds ratio in the prevalence of depression (OR = 2.5) among eleven industrial countries investigated when compared to men^64^. Our results could partly explain the higher prevalence of insomnia or psychiatric disorders in Japanese women, although further studies are required.

Our study has several limitations. One is selection bias, since almost all data stored in the Holter database were collected from subjects who were suspected of having some form of cardiovascular disease^21^, such as arrhythmia. Furthermore, the study may have included subjects with conditions that severely disturb sleep, such as depression, insomnia, and sleep apnea. Another potential source of selection bias was alcohol consumption or the use of medications such as hypnotics, both of which induce drowsiness and also affect sleep structure. Controlling for these effects might be necessary to obtain normative sleep values^1,5^ in the Japanese population.

Another limitation pertained to sleep-scoring algorithms. Our algorithms were developed and validated mainly for use in young, healthy adults^22^ (Supplementary Information) and were not fully evaluated in patients with sleep disorders or in the elderly. In addition, comparison with PSG was not performed. The lower sensitivity of the accelerometer for slight movements accompanied by micro-arousals during sleep is another concern; compared to wrist-worn wearable devices (e.g., actigraph), a chest-attached accelerometer is likely to fail to detect such movements. Chest movements induced mainly by snoring or abnormal breathing can slightly contaminate acceleration signals as noise and might affect the performance of sleep–wake classifications. In addition, this is a common limitation for acceleration-based sleep studies^30,32^, in which quiet wakefulness (e.g., the case where a subject reads a book or watches TV while lying in bed) tends to be scored as sleep, leading to a less accurate estimate of sleep and wake minutes.

## Conclusion

Our study utilized a large-scale acceleration database of 68,604 Japanese ranging from adolescents to the elderly and demonstrated the characteristic age- and gender-related differences in objective sleep properties assessed in real-life settings. Sleep durations estimated by TIB and TST increased in the elderly when compared to middle-aged adults, leading to a U-shaped association with aging. Japanese women slept shorter with lower sleep quality than men. This finding would be specific to the Japanese population and can be linked with several intricately intertwined variables, such as biological aging, socio-cultural, and socio-familial factors. In conclusion, our finding on age- and gender-association of sleep properties might contribute to the understanding of Japanese sleep habits.

## Supplementary Information

Supplementary Information.
