# Supplementary material for: Age and gender differences in objective sleep properties using large-scale body acceleration data in a Japanese population
Source: Sci Rep. 2021 May 11;11:9970. doi: 10.1038/s41598-021-89341-x (PMC8113448; doi:10.1038/s41598-021-89341-x)
Supplement: Supplementary file 1 — Supplementary Information. [file 41598_2021_89341_MOESM1_ESM.docx]

Supplementary Information

Age and gender differences in objective sleep properties using large-scale body acceleration data in a Japanese population

Li Li^1, 2, #^, Toru Nakamura^1, #, *^, Junichiro Hayano^3, *^, Yoshiharu Yamamoto^4, *^

^1^Graduate School of Engineering Science, Osaka University, 1-3 Machikaneyama, Toyonaka, Osaka 560-8531, Japan

^2^Intasect Communications, Inc., 3-1 Ogawa-cho, Kanda, Chiyoda-ku, Tokyo 101-0052, Japan

^3^Graduate School of Medical Sciences, Nagoya City University, 1 Kawasumi Mizuho-cho Mizuho-ku, Nagoya 467-8601, Japan

^4^Graduate School of Education, The University of Tokyo, 7-3-1 Hongo, Bunkyo-ku, Tokyo 113-0033, Japan

^#^ equally contributing authors

^*^ corresponding authors

Address correspondence to:

Toru Nakamura, PhD

Graduate School of Engineering Science, Osaka University

1-3 Machikaneyama, Toyonaka, Osaka 560-8531, Japan.

Tel +81- 6-6850-8258

Fax +81- 6-6850-8258

E-mail address: t-nakamura@sangaku.es.osaka-u.ac.jp

1. **Sleep–wake annotations**

To obtain objective sleep parameters from tri-axial trunk acceleration data measured by 24-hour Holter recording, we first constructed minute-by-minute sleep–wake classifiers based on machine learning approaches^1^; we conducted simultaneous measurements of 24-hour Holter monitoring and Actigraphy and then trained machines using the obtained data. Three machine learning algorithms (support vector machine (SVM), *k*-nearest neighbor (*k*-NN), and *k*-means clustering (*k*-means)) were utilized; local statistics (feature vectors) derived from Holter acceleration data were used as input data to the machines, and sleep–wake states derived from actigraphic data were used as supervised data. The performance of the constructed machines was compared. Using the classifier with the highest performance, we conducted minute-by-minute sleep–wake annotations and further scored sleep parameter values. The details are explained below.

- 1. **Subjects and experiments**

A total of 28 healthy adults (38.3 ± 15.3 years old, 26 males and 2 females) participated in the experiment. We conducted 24-hour simultaneous measurements of trunk acceleration data using a Holter recorder (Cardy 303 pico+, Suzuken Co., Ltd., Japan) and wrist activity data using an actigraph (Ambulatory Monitoring Inc., Ardsley, NY, USA) in daily life. All participants were instructed to wear an actigraph on the wrist of their nondominant hand at all times, except during rigorous exercise or bathing. The activity data during the period when the device was removed were identified and excluded from the analysis.

Written informed consent was obtained from all participants after they were fully informed about the purpose, procedures including risks and benefits of the study. This study was approved by the ethics committee of Osaka University and met the criteria of the declaration of Helsink.

**1-2 Sleep–wake evaluation by actigraphy**

The actigraph, which accumulates zero-crossing counts of wrist acceleration data every 1 minute, can evaluate minute-by-minute sleep–wake states accurately with more than 90% coincidence with polysomnography (PSG) testing by using the Cole-Kripke equation^2^. This equation works as a smoothing filter that forms a weighted sum of activity counts for the current minute, the previous 4 minutes, and the subsequent 2 minutes (i.e., a smoothing filter with a 7-minute window). If the filtered count at the current minute is greater than one, the current minute is labeled “wake,” otherwise it is labeled “sleep.” In this study, sleep–wake states determined by the Cole-Kripke equation were used as supervised data (binarized sequences indicating “sleep” and “wake”) to construct sleep–wake classifiers.

**1-3 Feature extraction and selection**

We extracted feature vectors characterizing local statistical properties of tri-axial trunk acceleration data. A flow chart of our feature extraction process is shown in Fig. S1. Each acceleration signal was resampled at 30 Hz; then, a low-pass filter was applied with a cutoff frequency of 0.25 Hz to separate the signal into a bodily motion component (BMC) and a gravitational component (GC)^3,4^. The former component is related to the amount of physical activity and the latter component provides information related to upper-body postures. The BMC signals were further filtered using a band-pass filter (2-3 Hz), as specified by the AMI actigraph^5^. The filtered BMC signals were converted into the signal magnitude area (SMA: $\mathrm{SMA}_{i}=\left| BMCx_{i} \right|+\left| {BMCy}_{i} \right|+\left| {BMCz}_{i} \right|$, where *BMCx_i_*, *BMCy_i_*, and *BMCz_i_* are the *i*th BMC signal data in the *x*-, *y*-, and *z*-directions, respectively). In addition, the trunk angles, ${\theta_{i}=cos}^{-1} (GCy_{i}/g )$ and ${\text{Φ}_{i}=cos}^{-1} ({GCz}_{i}/\sqrt{{GCx}_{i}^{2}+{GCz}_{i}^{2}} )$, were calculated from GC signals, where *g* was the acceleration due to gravity.

The resultant three signals (SMA, $\theta$, and $\text{Φ}$) were divided into 1-minute windows and basic statistics (mean, sum, standard deviation, variance) were then calculated for each window. In addition, the sum of the power spectrum amplitudes of BMC signals in the 2–3 Hz range in each window (${TPower}_{k}={Power\left( BMCx \right)}_{k}+{Power\left( BMCy \right)}_{k}+{Power\left( BMCz \right)}_{k}$, where *Power*(*BMCm*)*_k_* is the sum of the power spectrum amplitudes of the BMC signal in the *m*-direction in the *k*th window) was also calculated. The ${TPower}_{k}$ can be mathematically interpreted as the sum of the variance of band-pass-filtered BMC signals in each window^6^.

This resulted in total of 40 statistics (features) for each window. Factor analysis was applied to classify these features into subgroups of highly correlated features (results not shown). Since we found three distinctive subgroups by Factor analysis, we selected the following three statistics, *TPower*, mean trunk angles *θ*, and *φ*, as a representative feature for each subgroup. Then, we constructed classifiers using those features and evaluated their performance in minute-by-minute sleep–wake classification^1^. Following the Cole-Kripke algorithm, we created feature vectors consisting of the selected features from the previous 4 minutes and the subsequent 2 minutes (21 total features) for each window.

**1-4 Sleep–wake classifiers by machine learning approaches**

We used the following three machine learning approaches to construct sleep–wake classifiers and then compared their performance^1^.

***support vector machine* (*SVM*):** SVM is a supervised machine learning technique that is widely used for solving problems in classification, regression, and novelty detection. In two-class classification problems, SVM finds an optimal hyperplane separating given data points between two classes; an algorithm first transforms an input vector into higher-dimensional vectors via a kernel function and then constructs a linear hyperplane that maximizes the distance (“*margin*” of separation) between the two classes in the feature space. To search for the hyperplane maximizing the margin, SVM solves a quadratic programming problem. In practice, this primal optimization problem can be solved via its dual form^7,8^.

The construction of SVMs involves selecting a kernel function. The most commonly used functions are linear, polynomial, and Gaussian. Because of the lack of established criteria regarding the choice of the optimal kernel, we compared the performance of SVM classifiers constructed using each kernel. Since the Gaussian kernel, $K(\boldsymbol{x}, \boldsymbol{x}^{'}) =exp(-\gamma{\parallel\boldsymbol{x}-\boldsymbol{x}^{'}\parallel}^{2})$, demonstrated the highest performance in our test, we show only the results with this kernel.

***k-nearest neighbor* (*k*-NN):** The *k*-NN algorithm is a simple and low-computational-complexity method for pattern recognition^7,9^. The algorithm is referred to as “lazy learning” because it does not include a training phase. This algorithm stores the entire training data (or features) with their desired class. In the classification step, it retrieves the *k* least data points from the training dataset as neighbors of a given new data point, based on a similarity in the feature space. The class of the new data point is predicted based on the weighted majority class of the *k* nearest neighbors.

***k-means clustering* (*k-means*):** The *k*-means algorithm is one of the most popular unsupervised clustering methods for solving the problem of partitioning given data points into *k* disjoint clusters, based on minimizing a formal objective function^7^. For a given dataset consisting of *N* observations in *d*-dimensional space and an integer *k*, this algorithm finds a set of *k* points in *d*-dimensional space representing centers of clusters, and also assigns data points to the clusters to minimize the averaged square distance (e.g., Euclidean distance) of each data point to its nearest center. A well-known drawback of this method is that the clustering quality is extremely sensitive to the initialization. To compensate for this problem, we employed the *k*-means++ algorithm^10^, which obtains an initial set of centers that is probably close to the optimum solution.

***Training, validation, and testing*:** For the construction of SVM and *k*-NN classifiers, we randomly split the dataset into a training dataset (*N* = 18) and a test dataset (*N* = 10). We used the 21 features derived from acceleration signals for each window as input data, and used a sleep–wake estimate calculated by the Cole-Kripke equation from the actigraphic data as supervised data. The training dataset was used only for training processes, while the test dataset was used to evaluate each classifier’s performance. The stratified 10-fold cross-validation method was further employed to obtain optimal hyper-parameter values (the optimal hyperparameter values for the SVM model: regularization parameter *C* = 1.23, Gaussian kernel parameter $\gamma$ = 4.93; and for the *k*-NN model: number of neighbors *k* = 6).

For the *k*-means, we used the entire dataset for the classification and then evaluated its performance.

**1-5 Performance of sleep–wake classifiers**

The performance of the constructed classifiers was evaluated in terms of their accuracy, sensitivity, specificity, and F1-score^11^. These metrics were defined as follows; accuracy = (TP+TN)/(TP+FP+TN+FN), sensitivity = TP/(TP+FN), specificity = TN/(TN+FP), and F1-score = 2TP/(2TP+FP+FN), where TP represents the number of true positives, FP represents the number of false positives, TN represents the number of true negatives, and FN represents the number of false negatives. The F1-score is the harmonic average of sensitivity and specificity that provides a balanced measure of classification accuracy.

The performance of the constructed classifiers is summarized in Table S1. The SVM-based classifier showed the highest performance in all metrics (accuracy, 94.4 ± 3.8%; specificity, 94.2 ± 5.2%; sensitivity, 94.8 ± 3.9%; F1-score, 92.0 ± 4.5) except sensitivity score, which was highest with *k*-means. However, for the remaining metrics *k*-means demonstrated considerably worse performance than the other classifiers. We therefore selected the SVM-based classifier as the optimal model for the sleep–wake classifier. Figure S2 shows an example of sleep–wake estimates obtained using the SVM-based classifier; these estimates exhibit satisfactory coincidence with those obtained using the Cole-Kripke equation with actigraphic data.

**2. Automatic scoring of sleep parameters**

In order to score sleep parameters, we used the low-pass filtered trunk angle (*θ*) and *TPower* signals to develop an algorithm that automatically determined In-bed time (IBT; clock time when a subject got in bed to sleep and then switched the light off) and Get-up time (GUT; clock time when a subject finally awakened in the morning). The material below summarizes the detection algorithms for IBT and GUT, together with the validation results of our sleep parameter scoring.

**2-1 Total variation denoising**

Total variation (TV)-based filtering was applied to trunk-tilt angle data (*θ*). TV-based filtering was initially introduced for image restoration^12^. This filter provides a piecewise-constant solution for smooth regions contained in a given image, with preserving image edges. This filtering was also applied to one-dimensional signals as an effective method for denoising signals that are approximated by piecewise constants^13-15^. The trunk angle data reflecting upper-body tilt angles could be well approximated by a constant value during the period when a subject was in a steady state (e.g., quiet standing, sitting, or lying). Therefore, TV denoising is one possible choice for detecting periods of lying in bed.

The TV of a signal *x*(*t*) is defined as the summation of absolute difference values between time *t* and *t*+1: $\mathrm{TV}\left( x \right)=\sum_{t=1}^{N-1} \left| x\left( t+1 \right)-x\left( t \right) \right|$. For a given signal *y*, the TV denoising method finds the signal *x* that minimizes the objective function: *J*$\left( x \right)=\sum_{t=1}^{N-1} \left( y\left( t \right)-x\left( t \right) \right)^{2}+\lambda\mathrm{TV}\left( x \right)$. The regulation parameter $\lambda$ controls the amount of denoising or the smoothness of the signal *x*. With an increase in the parameter value of $\lambda$, the output solution becomes more nearly piecewise constant. In this study, the parameter value of $\lambda$ was set to 5.0. The optimal numerical solution for TV filtering was obtained using the algorithm proposed by A. Chambolle^16^.

Figure S3 compares the results of filtered trunk angle (*θ*) signals by TV denoising filtering and Butterworth filtering. Compared with Butterworth filtering, TV denoising filtering can approximate the trunk angle signal by piecewise-constant values, enabling us to segment upper-body postures at higher time resolution and also to trace rapid changes in trunk angles associated with postural transitions, such as from sitting to lying.

**2-2 Get-up time and In-bed time detection**

The algorithm for detecting get-up time (GUT) and in-bed time (IBT) is as follows: a median filter with a 7-point window was applied to a minute-by-minute trunk tilt angle signal $\theta\left( t \right)$, and then TV denoising filtering was performed. The filtered signal was rescaled to [0, 1] to reduce individual variations. If the value of the rescaled signal at time *t* was smaller than 0.6, the state at time *t* was labeled “lying,” otherwise it was labeled “not lying.” This threshold value corresponded to a mean trunk tilt angle of 128.3 ± 3.4 degrees across subjects. Validation of the “lying” label on a minute-by-minute basis was conducted using the *Tpower* signal passed through a median filter with an 11-point window. If the value of the *Tpower* signal at time *t* was greater than 0.02, the label was set to “not lying.” Based on the label sequence, we searched for the longest lying period to determine major sleep^17^. The start and end times of major sleep were regarded as GUT and IBT, respectively. The threshold values used in the algorithm were determined by a grid search method using the training samples (*N*=18).

**2-3 Sleep parameters**

In accordance with the definitions of sleep parameters used by the analytic software (Action-W2; AW2)^17^ associated with AMI actigraphic devices, we scored additional five sleep parameters: time-in-bed (TIB, duration a subject spent in bed), sleep latency (SL, number of minutes until the first of 10 consecutive minutes of sleep following IBT), wake after sleep onset (WASO, the total wake time, in minutes, from sleep onset to the Get-up time), total sleep time (TST, TIB minus SL and WASO), and sleep efficiency (SE, TST divided by the sleep period [“O–O interval”^17^] times 100). These parameters were also scored from the actigraphic data by the software as the ground truth values for validation of our algorithm. Note that as recommended elsewhere^18^, we applied Webster’s five rules of rescoring to sleep–wake estimates before scoring the sleep parameters.

It is important to address the accuracy of actigraphy-based sleep parameters. A recent systematic review and meta-analysis^19^ concluded that actigraphy overestimated TST and SE compared to PSG in healthy adults, but not with significance. On the other hand, actigraphy significantly underestimated SL by 8.1 minutes relative to PSG. WASO was also nonsignificant underestimated by actigraphy. These biases might stem from the limited capacity of actigraphy to identify waking periods during sleep^19,20^.

**2-4 Bland-Altman analysis**

The Bland-Altman concordance technique^21^ was utilized to examine degree of agreement between AW2 and our algorithm. This technique plots differences in sleep parameter values scored by two methods against the mean values of the two methods (Fig. S4). The statistical significance of the mean difference from the zero level indicates the existence of a negative or positive bias; a significant negative bias indicates that our algorithm tends to overestimate a particular sleep parameter compared with AW2, while a positive bias indicates that the sleep parameter is underestimated. The statistical significance of the mean difference from zero was tested with the *t*-test. Proportional bias was further examined by regression analysis. The significant slope of the regression line indicates that the difference between the two methods proportionally changes according to the mean level of the sleep parameter values.

Figure S4 shows Bland-Altman plots of each sleep parameter. In two subjects, we found large deviations of sleep parameter values between AW2 and our method (the difference in the IBT was 82 minutes in one subject and 127 minutes in the other). These subjects used a smartphone while lying in bed before they fell asleep. Such “quiet wakefulness” in a lying position possibly caused the discrepancies in sleep parameter scoring.

In Fig. S4, we plot the mean differences and 95% limits of agreement when the two outlier samples were excluded from the analysis. Our method overestimated IBT by 2.8 minutes and WASO by 8.8 minutes, and underestimated TST by 12.3 minutes, GUT by 1.4 minutes, TIB by 3.1 minutes, SE by 1.8%, and SL by 1.4 minutes. However, none of these differences were significant (*p* > 0.05). This indicates that the sleep parameter scores obtained by our method have good coincidence with those by AW2.

Notably, even though the two outlier samples were included in the analysis, the differences of the two methods were not significant for any of the sleep parameters; the mean differences were 32.9 minutes for TST, 23.1 minutes for IBT, 0.6 minutes for GUT, 22.7 minutes for TIB, 2.1% for SE, 1.0 minutes for SL, and 9.3 minutes for WASO.

We also conducted the regression analysis under two conditions, namely including and excluding the two outlier samples. We did not confirm any downward or upward trend for any sleep parameter in either condition.

**3. GLM results with different reference groups**

Table S3 summarize the results of statistical test by GLMs with different reference groups for each sleep parameter. In the Table, a reference group was changed from age of 10s to 80s. Each cell in the table lists age groups that were significant from a reference group.

***
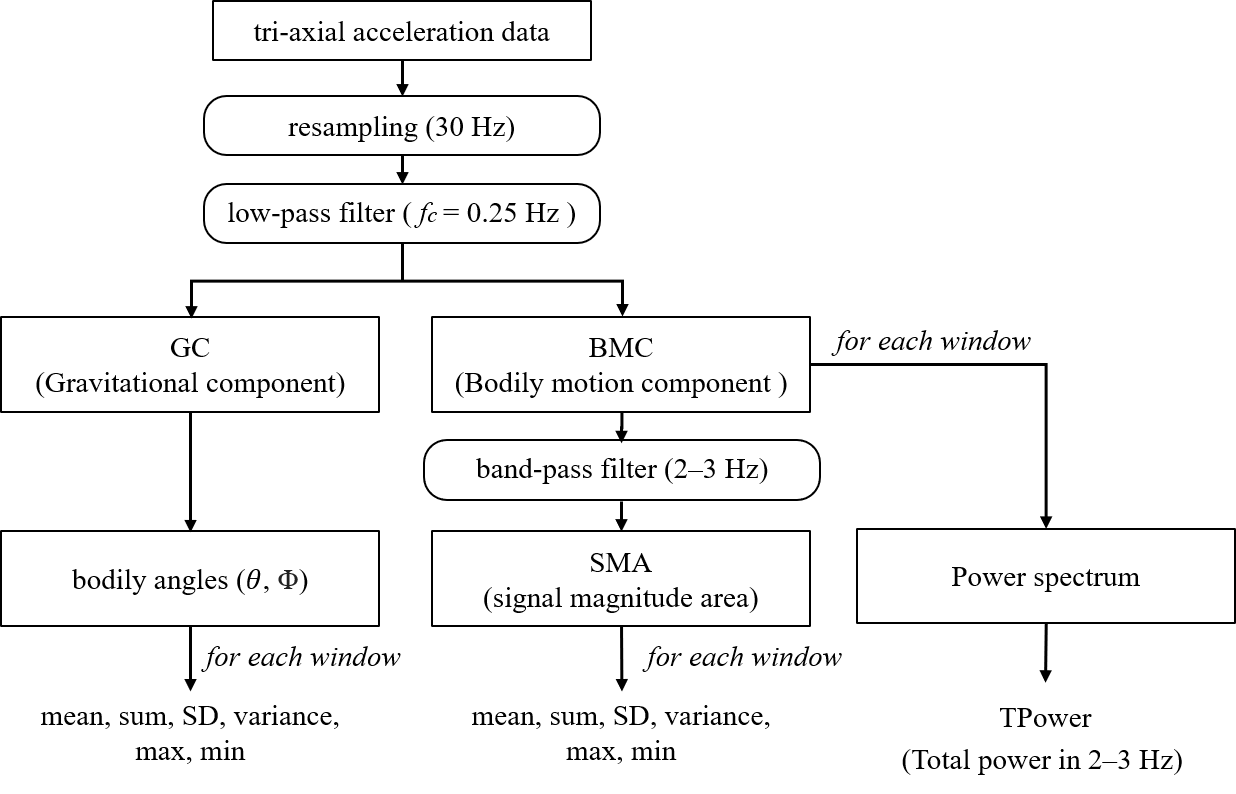
***

Figure S1. A flow chart summarizing the extraction of feature vectors from tri-axial acceleration data.

**
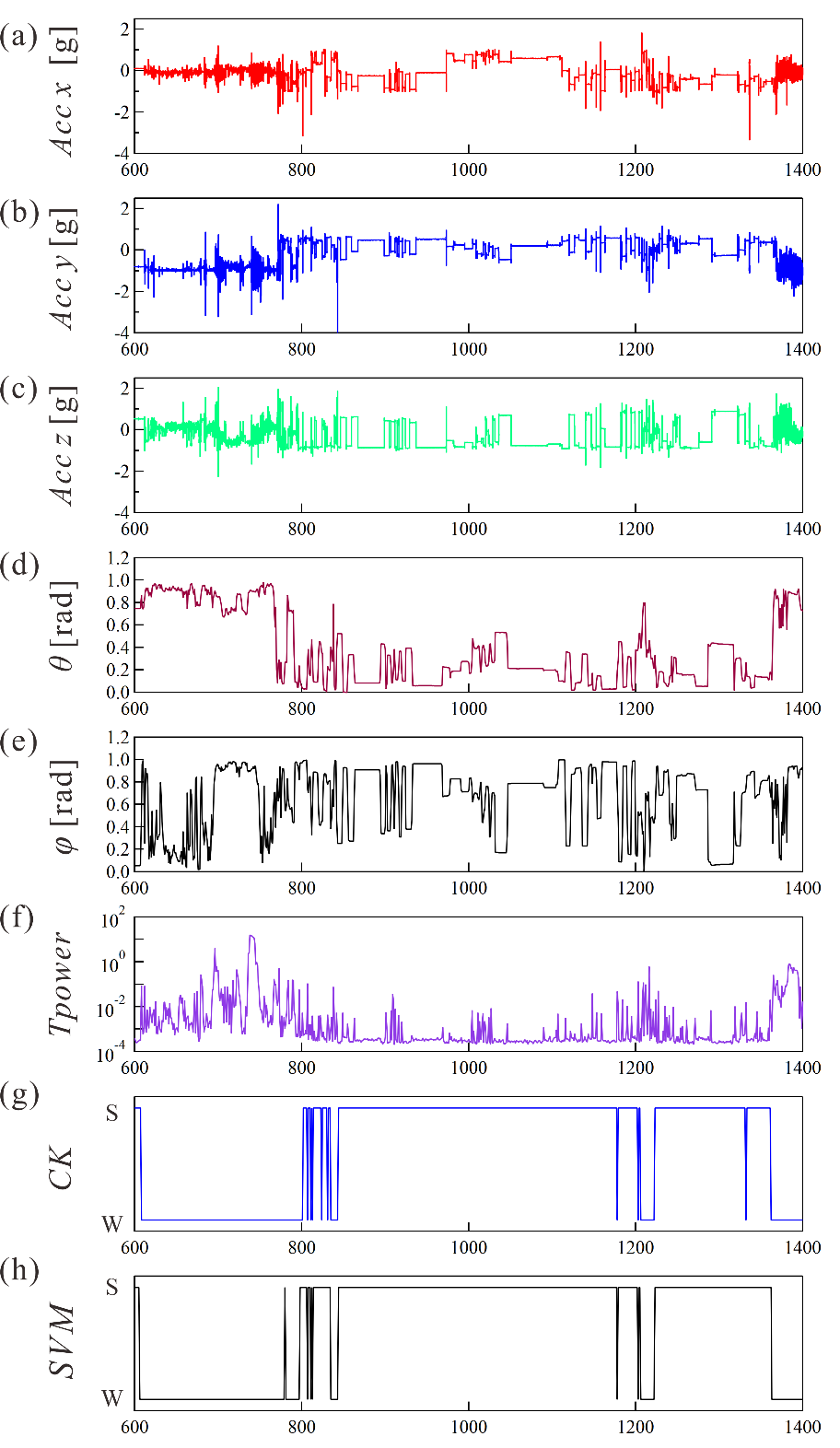
**

Figure S2. An example of feature signals extracted from trunk tri-axial acceleration data and estimates of sleep–wake states by the constructed SVM-based classifier. (a)(b)(c) Trunk acceleration data measured by a Holter recorder over 800 minutes. *Acc x*, *Acc y*, and *Acc z* represent acceleration data in the medio–lateral, antero–posterior, and vertical direction, respectively. The sequences of minute-by-minute averaged trunk angles (d) *θ* and (e) *φ* and (f) *TPower* are shown. Note that the vertical axis of *TPower* is shown on the logarithmic scale for illustration purposes. (g) Sleep–wake states estimated by the Cole-Kripke equation (*CK*) and (h) those estimated by the classifier (*SVM*). The labels “S” and “W” in the vertical axes in (g) and (h) indicate the “sleep” and “wake” states, respectively.


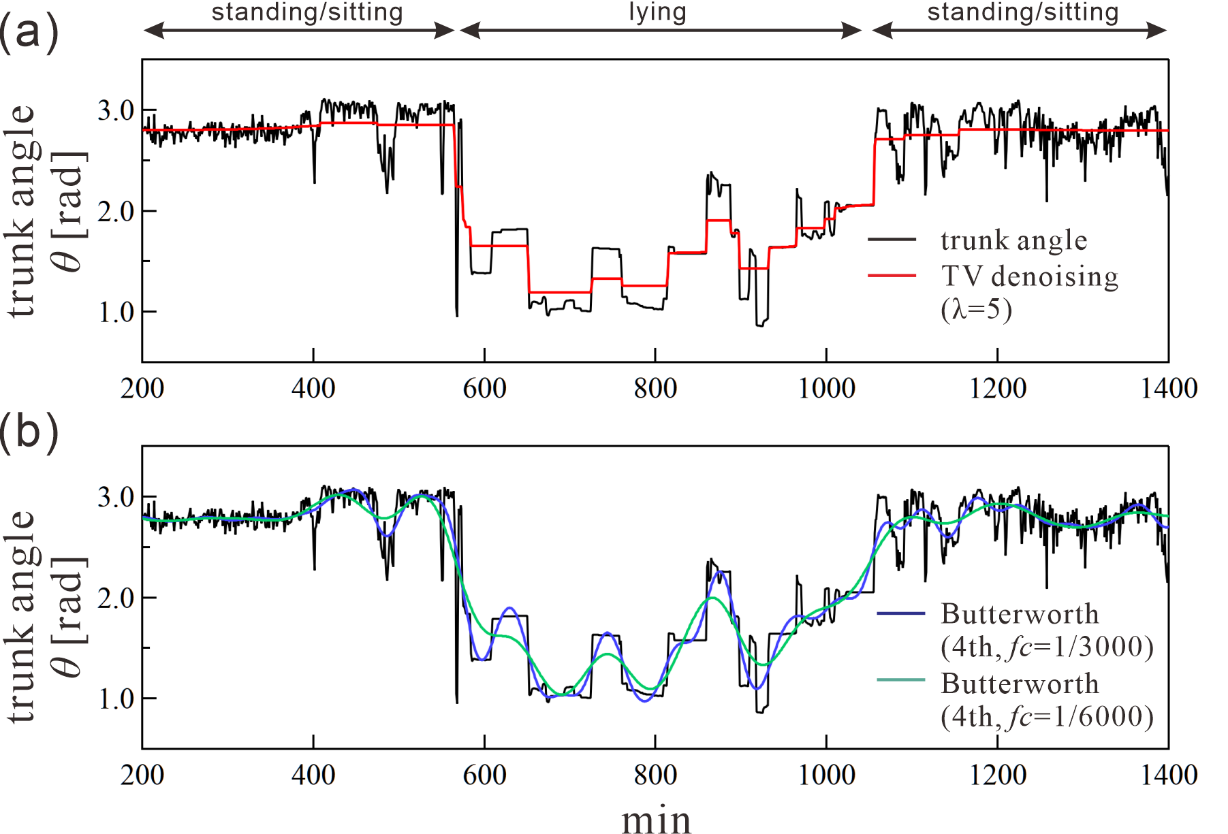


Figure S3. A comparison of filtered trunk angle data by TV denoising filtering and Butterworth filtering. (a) The results of TV denoising; the black curve shows the trunk tilt angle signal (*θ*) and the red curve shows the TV-filtered data. (b) The results of 4th order Butterworth filtering with different cut-off frequencies *fc* (blue, *fc* = 1/3000 Hz; green, *fc* = 1/60000 Hz).

**
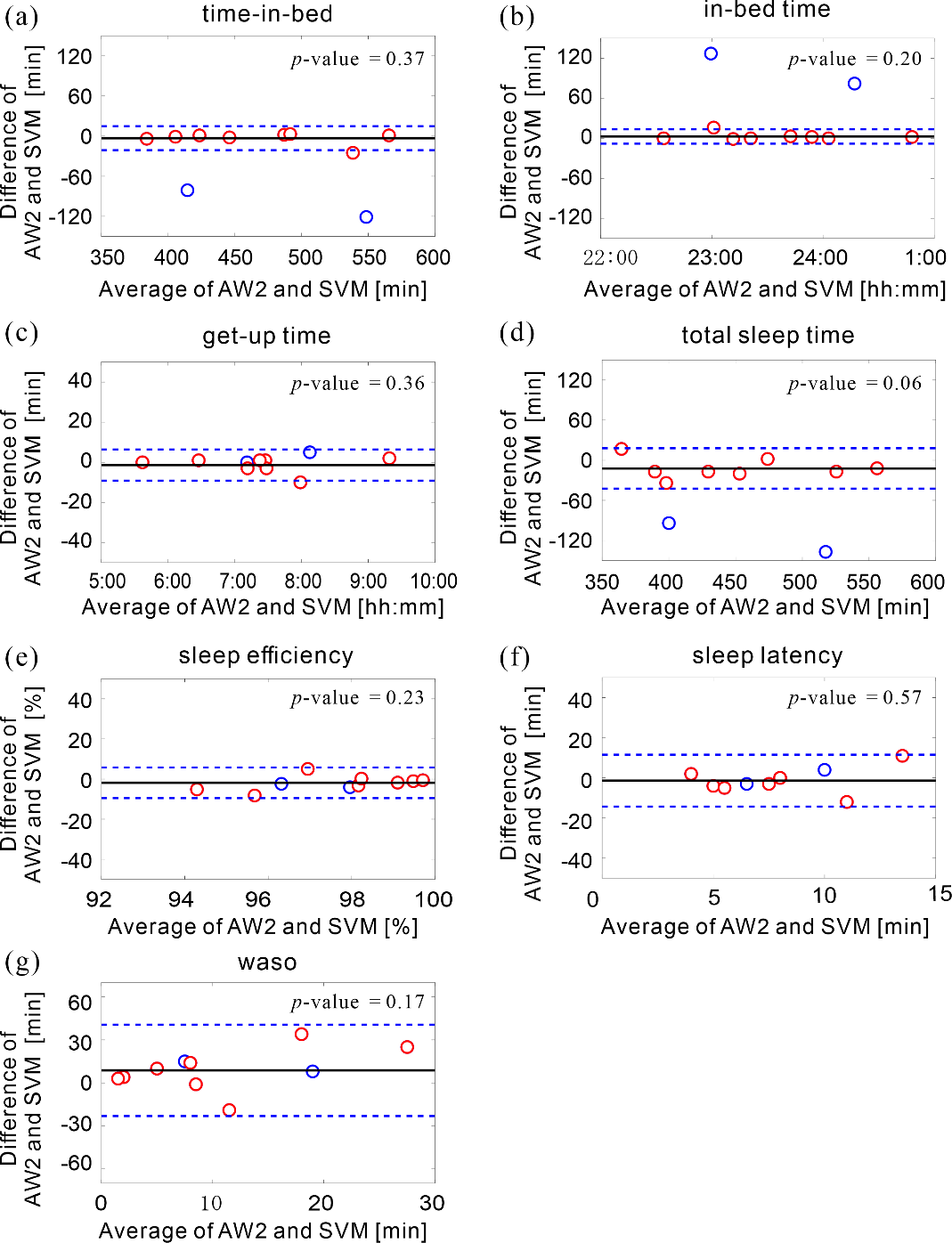
**

Figure S4. Bland-Altman plots demonstrating differences in sleep parameters between an AMI Actigraph (AW2 software) and our algorithm. (a) time-in-bed, (b) in-bed time, (c) get-up time, (d) total sleep time, (e) sleep efficiency, (f) sleep latency, and (g) WASO. The sleep parameter values of two outlier samples are plotted using blue circles. The solid horizontal line (black) in each panel represents the mean level of the differences between AW2 and our algorithm (AW2 minus SVM). The broken lines (blue) indicate the limits of agreement (± 1.96 SD). Each *p*-value indicates the result of the *t*-test of equality of mean differences from zero between the two methods.


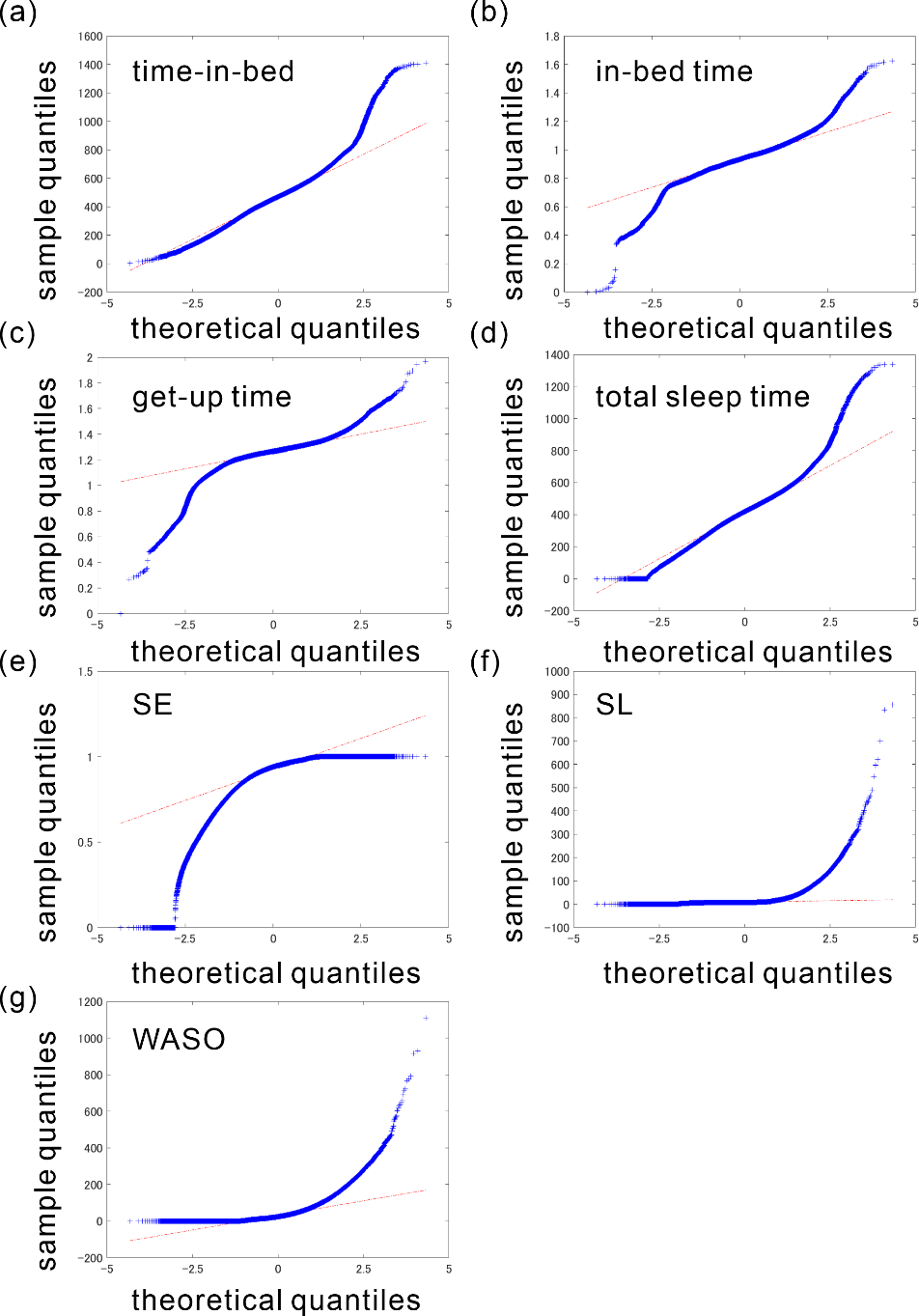


Figure S5. Q-Q plots for sleep parameters. (a) time-in-bed, (b) in-bed time, (c) get-up time, (d) total sleep time, (e) sleep efficiency, (f) sleep latency, and (g) WASO.


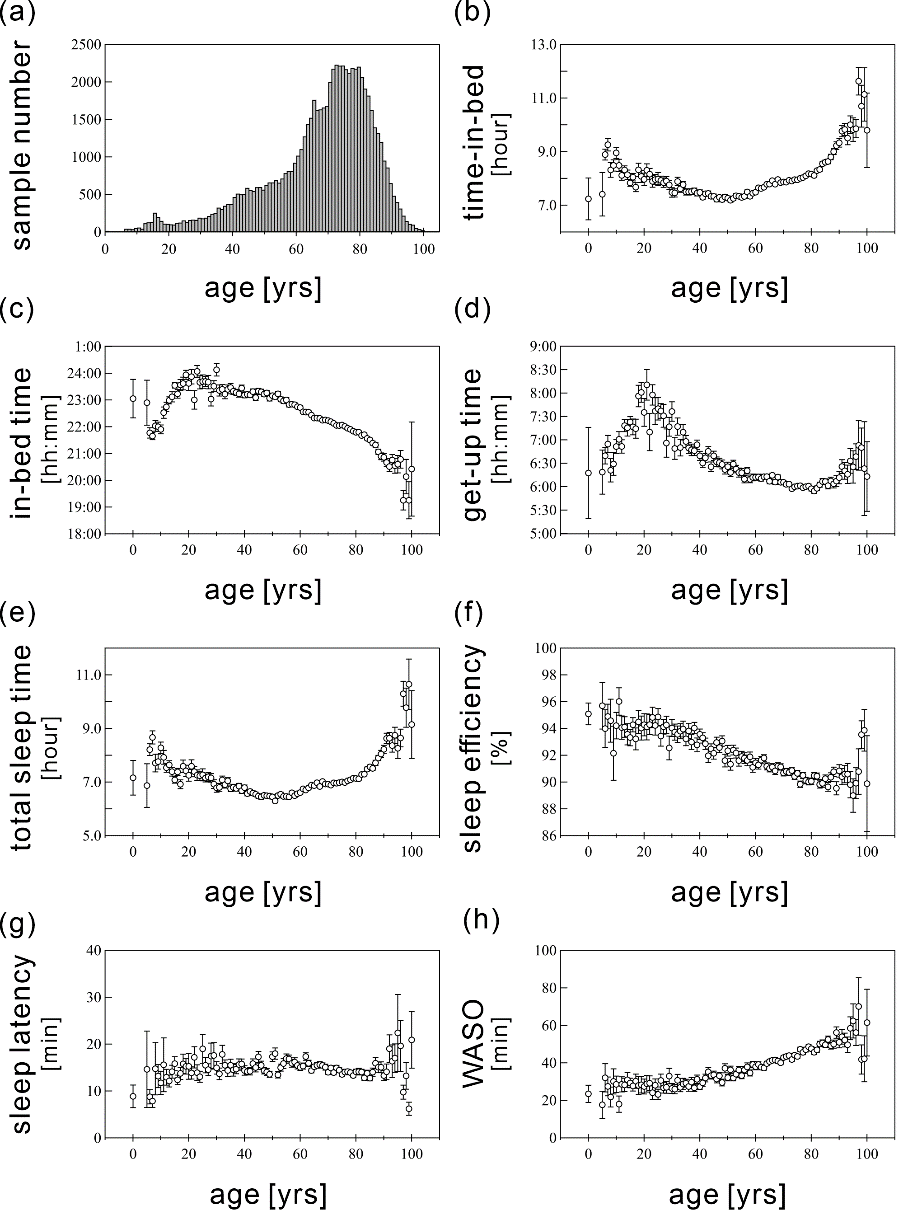


Figure S6. Age distribution and age differences in sleep parameters: (a) The age distribution with bin-width of 1 year old. (b-h) The mean values of sleep parameter as a function of the continuous variable of age; (b) time-in-bed, (c) in-bed time, (d) get-up time, (e) total sleep time, (f) sleep efficiency, (g) sleep latency, and (h) wake time after sleep onset (WASO). The error bars indicate the standard error of the mean. Note that data with less than 10 samples was not plotted in each panel because of their inaccuracy.


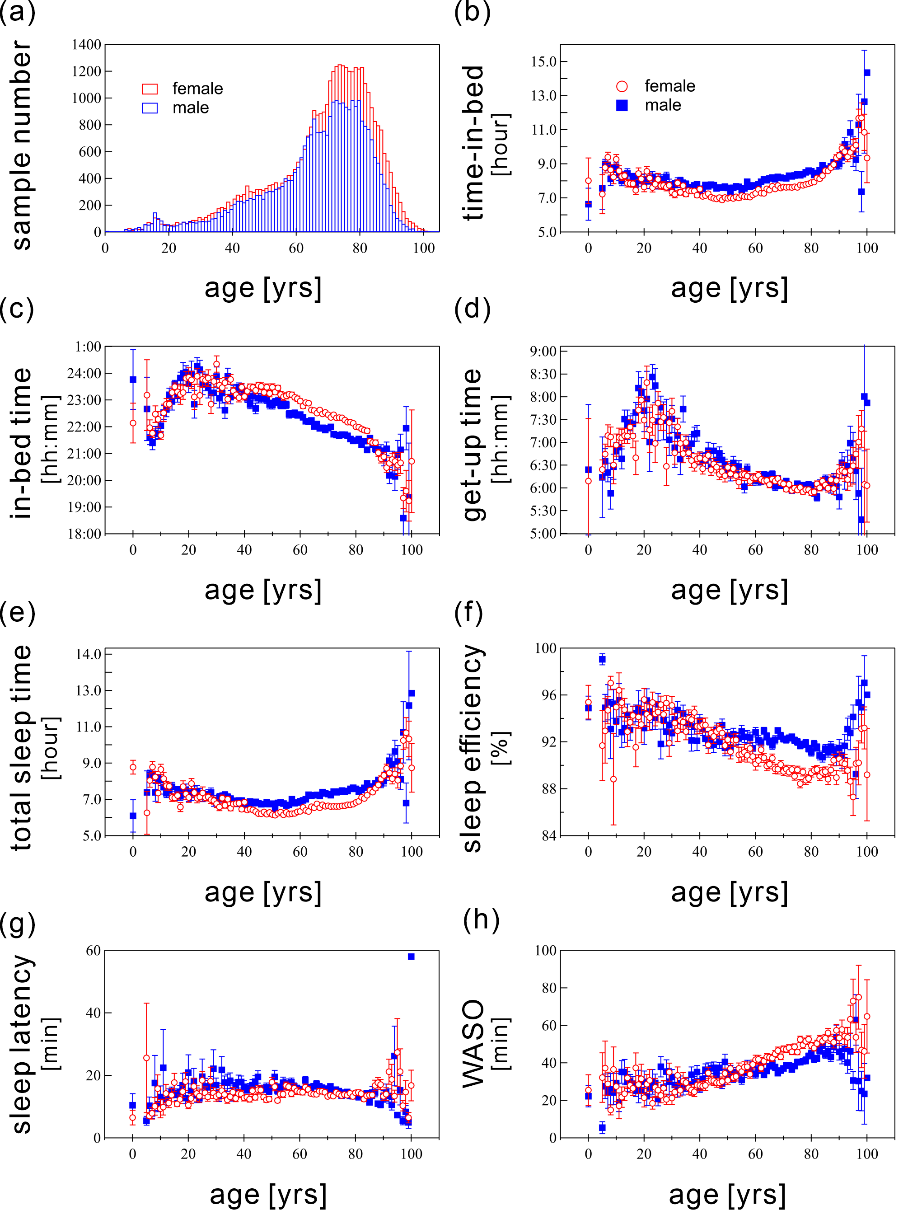


Figure S7. Gender differences in age distributions and sleep parameter values: (a) the sample’ age distribution stratified by gender. (b-h) the mean values of sleep parameter as a function of the continuous variable of age; (b) time-in-bed, (c) in-bed time, (d) get-up time, (e) total sleep time, (f) sleep efficiency, (g) sleep latency, and (h) wake time after sleep onset (WASO). The error bars indicate the standard error of the mean. Note that data with less than 10 samples was not plotted in each panel because of their inaccuracy.

Table S1: Classification performance of constructed models.

|  | accuracy [%] | specificity [%] | sensitivity [%] | F1-score |
| --- | --- | --- | --- | --- |
| SVM | 94.4 ± 3.8 | 94.2 ± 5.2 | 94.8 ± 3.9 | 92.0 ± 4.5 |
| *k*-NN | 92.1 ± 4.3^*^ | 91.8 ± 5.7^*^ | 92.8 ± 4.1^*^ | 88.8 ± 5.2^*^ |
| *k*-means | 37.7 ± 7.4^†^ | 1.1 ± 2.4^†^ | 100 ± 0.0^†^ | 56.9 ± 7.8^†^ |

Values are represented as mean ± SD. The performance metrics were calculated from the test dataset (*N* = 10) for the supervised methods (SVM and *k*-NN), and from the entire dataset (*N* = 28) for the unsupervised method (*k*-means).

^*^, significant difference between SVM and *k*-NN (*p* < 0.01; paired *t*-test).

^†^, significant difference between SVM and *k*-means (*p* < 0.01; *t*-test).

Table S2: Mean and standard error of mean for sleep parameters stratified by age group and gender

| Age group | Strata by gender | TIB | In-bed time | Get-up time | TST | SE | SL | WASO |
| --- | --- | --- | --- | --- | --- | --- | --- | --- |
|  | (sample number) | [min] | [hh:mm] | [hh:mm] | [min] | [%] | [min] | [min] |
| 10s | total (1,314) | 485.2 ± 3.4 | 23:16 ± 0:03 | 7:21 ± 0:03 | 443.4 ± 3.3 | 93.9 ± 0.2 | 14.0 ± 0.5 | 28.6 ± 1.2 |
|  | male (684) | 487.1 ± 4.9 | 23:20 ± 0:04 | 7:27 ± 0:04 | 445.0 ± 4.6 | 93.9 ± 0.3 | 14.3 ± 0.8 | 29.2 ± 1.6 |
|  | female (625) | 483.1 ± 4.8 | 23:33 ± 0:06 | 7:23 ± 0:06 | 441.7 ± 4.8 | 93.9 ± 0.4 | 13.7 ± 0.8 | 27.9 ± 1.7 |
| 20s | total (1,421) | 476.7 ± 3.7 | 23:34 ± 0:04 | 7:31 ± 0:05 | 432.9 ± 3.5 | 94.0 ± 0.2 | 15.8 ± 0.7 | 28.0 ± 1.1 |
|  | male (655) | 482.8 ± 6.0 | 23:37 ± 0:07 | 7:40 ± 0:07 | 435.7 ± 5.5 | 93.8 ± 0.4 | 17.1 ± 1.2 | 29.5 ± 1.8 |
|  | female (761) | 470.5 ± 4.6 | 23:33 ± 0:06 | 7:23 ± 0:06 | 430.2 ± 4.4 | 94.4 ± 0.3 | 14.7 ± 0.8 | 26.2 ± 1.4 |
| 30s | total (2,816) | 453.5 ± 2.4 | 23:24 ± 0:02 | 6:57 ± 0:02 | 409.9 ± 2.2 | 93.5 ± 0.2 | 15.3 ± 0.4 | 28.5 ± 0.8 |
|  | male (1,205) | 465.8 ± 3.8 | 23:20 ± 0:04 | 7:06 ± 0:04 | 418.4 ± 3.5 | 93.3 ± 0.3 | 17.3 ± 0.8 | 30.3 ± 1.2 |
|  | female (1,602) | 444.1 ± 3.0 | 23:26 ± 0:03 | 6:50 ± 0:03 | 403.6 ± 2.9 | 93.8 ± 0.2 | 13.7 ± 0.4 | 27.0 ± 1.0 |
| 40s | total (5,448) | 439.7 ± 1.7 | 22:24 ± 0:01 | 6:10 ± 0:00 | 392.9 ± 1.6 | 92.6 ± 0.1 | 14.8 ± 0.3 | 32.3 ± 0.6 |
|  | male (2,444) | 458.1 ± 2.7 | 23:01 ± 0:03 | 6:39 ± 0:03 | 407.1 ± 2.5 | 92.5 ± 0.2 | 16.5 ± 0.5 | 34.7 ± 1.0 |
|  | female (2,990) | 424.5 ± 2.1 | 23:23 ± 0:02 | 6:28 ± 0:02 | 381.1 ± 2.0 | 92.7 ± 0.2 | 13.5 ± 0.3 | 30.4 ± 0.8 |
| 50s | total (7,361) | 440.2 ± 1.5 | 22:58 ± 0:01 | 6:18 ± 0:01 | 389.2 ± 1.4 | 91.8 ± 0.1 | 16.1 ± 0.3 | 35.4 ± 0.6 |
|  | male (3,536) | 458.1 ± 2.3 | 22:41 ± 0:02 | 6:19 ± 0:02 | 406.2 ± 2.1 | 92.3 ± 0.2 | 17.0 ± 0.4 | 35.2 ± 0.8 |
|  | female (3,804) | 423.5 ± 1.9 | 23:13 ± 0:02 | 6:17 ± 0:01 | 373.4 ± 1.8 | 91.3 ± 0.2 | 15.2 ± 0.4 | 35.6 ± 0.8 |
| 60s | total (14,728) | 466.2 ± 1.1 | 22:24 ± 0:01 | 6:10 ± 0:00 | 411.2 ± 1.0 | 91.2 ± 0.1 | 15.5 ± 0.2 | 39.8 ± 0.4 |
|  | male (6,956) | 484.0 ± 1.7 | 22:07 ± 0:01 | 6:11 ± 0:01 | 431.3 ± 1.5 | 92.4 ± 0.1 | 16.3 ± 0.3 | 36.5 ± 0.5 |
|  | female (7,728) | 450.2 ± 1.3 | 22:40 ± 0:01 | 6:10 ± 0:01 | 393.2 ± 1.3 | 90.2 ± 0.1 | 14.7 ± 0.3 | 42.8 ± 0.6 |
| 70s | total (21,710) | 478.6 ± 1.0 | 22:02 ± 0:00 | 6:01 ± 0:00 | 419.9 ± 0.9 | 90.5 ± 0.1 | 14.1 ± 0.2 | 44.8 ± 0.4 |
|  | male (9,517) | 500.5 ± 1.5 | 21:43 ± 0:01 | 6:03 ± 0:01 | 446.2 ± 1.4 | 92.0 ± 0.1 | 14.6 ± 0.3 | 39.6 ± 0.5 |
|  | female (12,156) | 461.4 ± 1.2 | 22:18 ± 0:01 | 5:59 ± 0:01 | 399.1 ± 1.1 | 89.2 ± 0.1 | 13.8 ± 0.2 | 48.9 ± 0.5 |
| 80s | total (13,806) | 507.7 ± 1.5 | 21:34 ± 0:01 | 6:01 ± 0:01 | 444.7 ± 1.4 | 90.1 ± 0.1 | 14.0 ± 0.2 | 49.1 ± 0.5 |
|  | male (5,488) | 518.5 ± 2.4 | 21:23 ± 0:01 | 6:01 ± 0:01 | 460.0 ± 2.2 | 91.3 ± 0.1 | 13.3 ± 0.3 | 45.3 ± 0.7 |
|  | female (8,285) | 500.6 ± 1.9 | 21:41 ± 0:01 | 6:02 ± 0:01 | 434.6 ± 1.8 | 89.4 ± 0.1 | 14.5 ± 0.3 | 51.7 ± 0.7 |

Values are represented as mean ± SEM.

Table S3: List of significant age groups for sleep parameters.

| Reference  age group | Strata by gender  (sample number) | TIB | In-bed time | Get-up time | TST | SE | SL | WASO |
| --- | --- | --- | --- | --- | --- | --- | --- | --- |
| 10s | total (1,314) | 30s, 40s, 50s, 60s, 80s | 20s, 50s, 60s, 70s, 80s | 30s, 40s, 50s, 60s, 70s, 80s | 30s, 40s, 50s, 60s, 70s | 40s, 50s, 60s, 70s, 80s | 20s, 30s, 50s, 60s | 40s, 50s, 60s, 70s, 80s |
|  | male (684) | 40s, 50s, 80s | 50s, 60s, 70s, 80s | 40s, 50s, 60s, 70s, 80s | 30s, 40s, 50s | 40s, 50s, 60s, 70s, 80s | 20s, 30s, 40s, 50s | 40s, 50s, 60s, 70s, 80s |
|  | female (625) | 30s, 40s, 50s, 60s, 70s | 60s, 70s, 80s | 30s, 40s, 50s, 60s, 70s, 80s | 30s, 40s, 50s, 60s, 70s | 50s, 60s, 70s, 80s |  | 50s, 60s, 70s, 80s |
| 20s | total (1,421) | 30s, 40s, 50s, 80s | 10s, 40s, 50s, 60s, 70s, 80s | 30s, 40s, 50s, 60s, 70s, 80s | 30s, 40s, 50s, 60s, 80s | 40s, 50s, 60s, 70s, 80s | 70s, 80s | 40s, 50s, 60s, 70s, 80s |
|  | male (655) | 40s, 50s, 80s | 40s, 50s, 60s, 70s, 80s | 30s, 40s, 50s, 60s, 70s, 80s | 40s, 50s, 80s | 70s, 80s | 10s, 70s, 80s | 60s, 70s, 80s |
|  | female (761) | 30s, 40s, 50s, 60s, 80s | 60s, 70s, 80s | 30s, 40s, 50s, 60s, 70s, 80s | 30s, 40s, 50s, 60s, 70s | 40s, 50s, 60s, 70s, 80s |  | 50s, 60s, 70s, 80s |
| 30s | total (2,816) | 10s, 20s, 40s, 50s, 60s, 70s, 80s | 50s, 60s, 70s, 80s | 10s, 20s, 40s, 50s, 60s, 70s, 80s | 10s, 20s, 40s, 50s, 70s, 80s | 40s, 50s, 60s, 70s, 80s | 10s, 70s, 80s | 40s, 50s, 60s, 70s, 80s |
|  | male (1,205) | 60s, 70s, 80s | 40s, 50s, 60s, 70s, 80s | 20s, 40s, 50s, 60s, 70s, 80s | 10s, 70s, 80s | 80s | 10s, 70s, 80s | 50s, 60s, 70s, 80s |
|  | female (1,602) | 10s, 20s, 40s, 50s, 70s, 80s | 60s, 70s, 80s | 20s, 40s, 50s, 60s, 70s, 80s | 10s, 20s, 40s, 50s, 80s | 50s, 60s, 70s, 80s |  | 50s, 60s, 70s, 80s |
| 40s | total (5,448) | 10s, 20s, 30s, 60s, 70s, 80s | 20s, 50s, 60s, 70s, 80s | 10s, 20s, 30s, 50s, 60s, 70s, 80s | 10s, 20s, 30s, 60s, 70s, 80s | 10s, 20s, 50s, 60s, 70s, 80s | 50s, 70s, 80s | 10s, 20s, 30s, 50s, 60s, 70s, 80s |
|  | male (2,444) | 10s, 20s, 60s, 70s, 80s | 30s, 50s, 60s, 70s, 80s | 10s, 20s, 30s, 50s, 60s, 70s, 80s | 10s, 20s, 60s, 70s, 80s | 10s, 80s | 10s, 70s, 80s | 10s, 70s, 80s |
|  | female (2,990) | 10s, 20s, 30s, 60s, 70s, 80s | 60s, 70s, 80s | 10s, 20s, 30s, 60s, 70s, 80s | 10s, 20s, 30s, 60s, 70s, 80s | 20s, 50s, 60s, 70s, 80s | 50s, 60s, 80s | 50s, 60s, 70s, 80s |

Table S3 (continued): List of significant age groups for sleep parameters.

| Reference  age group | Strata by gender  (sample number) | TIB | In-bed time | Get-up time | TST | SE | SL | WASO |
| --- | --- | --- | --- | --- | --- | --- | --- | --- |
| 50s | total (7,361) | 10s, 20s, 30s, 60s, 70s, 80s | 10s, 20s, 30s, 40s, 60s, 70s, 80s | 10s, 20s, 30s, 40s, 60s, 70s, 80s | 10s, 20s, 30s, 60s, 70s, 80s | 10s, 20s, 30s, 40s, 60s, 70s, 80s | 10s, 40s, 70s, 80s | 10s, 20s, 30s, 40s, 60s, 70s, 80s |
|  | male (3,536) | 10s, 20s, 60s, 70s, 80s | 10s, 20s, 30s, 40s, 60s, 70s, 80s | 10s, 20s, 30s, 40s, 70s, 80s | 10s, 20s, 70s, 80s | 10s, 80s | 10s, 70s, 80s | 10s, 30s, 70s, 80s |
|  | female (3,804) | 10s, 20s, 30s, 60s, 70s, 80s | 60s, 70s, 80s | 10s, 20s, 30s, 70s, 80s | 10s, 20s, 30s, 60s, 70s, 80s | 10s, 20s, 30s, 40s, 60s, 70s, 80s | 40s, 70s | 10s, 20s, 30s, 40s, 60s, 70s, 80s |
| 60s | total (14,728) | 10s, 30s, 40s, 50s, 70s, 80s | 10s, 20s, 30s, 40s, 50s, 70s, 80s | 10s, 20s, 30s, 40s, 50s, 70s, 80s | 10s, 20s, 40s, 50s, 70s, 80s | 10s, 20s, 30s, 40s, 50s, 70s, 80s | 10s, 70s, 80s | 10s, 20s, 30s, 40s, 50s, 70s, 80s |
|  | male (6,956) | 30s, 40s, 50s, 70s, 80s | 10s, 20s, 30s, 40s, 50s, 70s, 80s | 10s, 20s, 30s, 40s | 40s, 50s, 70s, 80s | 10s, 80s | 70s, 80s | 10s, 20s, 30s, 70s, 80s |
|  | female (7,728) | 10s, 20s, 40s, 50s, 70s, 80s | 10s, 20s, 30s, 40s, 50s, 70s, 80s | 10s, 20s, 30s, 40s, 70s | 10s, 20s, 40s, 50s, 80s | 10s, 20s, 30s, 40s, 50s, 70s, 80s | 40s, 70s | 10s, 20s, 30s, 40s, 50s, 70s, 80s |
| 70s | total (21,710) | 30s, 40s, 50s, 60s, 80s | 10s, 20s, 30s, 40s, 50s, 60s, 80s | 10s, 20s, 30s, 40s, 50s, 60s | 10s, 30s, 40s, 50s, 60s, 80s | 10s, 20s, 30s, 40s, 50s, 60s | 20s, 30s, 40s, 50s, 60s | 10s, 20s, 30s, 40s, 50s, 60s, 80s |
|  | male (9,517) | 30s, 40s, 50s, 60s, 80s | 10s, 20s, 30s, 40s, 50s, 60s, 80s | 10s, 20s, 30s, 40s, 50 | 30s, 40s, 50s, 60s, 80s | 10s, 20s, 80s | 20s, 30s, 40s, 50s, 60s, 80s | 10s, 20s, 30s, 40s, 50s, 60s, 80s |
|  | female (12,156) | 10s, 30s, 40s, 50s, 60s, 80s | 10s, 20s, 30s, 40s, 50s, 60s, 80s | 10s, 20s, 30s, 40s, 50s, 60s | 10s, 20s, 40s, 50s, 80s | 10s, 20s, 30s, 40s, 50s, 60s | 50s, 60s, 80s | 10s, 20s, 30s, 40s, 50s, 60s, 80s |
| 80s | total (13,806) | 10s, 20s, 30s, 40s, 50s, 60s, 70s | 10s, 20s, 30s, 40s, 50s, 60s, 70s | 10s, 20s, 30s, 40s, 50s, 60s | 20s, 30s, 40s, 50s, 60s, 70s | 10s, 20s, 30s, 40s, 50s, 60s | 20s, 30s, 40s, 50s, 60s | 10s, 20s, 30s, 40s, 50s, 60s70s |
|  | male (5,488) | 10s, 20s, 30s, 40s, 50s, ,60s, 70s | 10s, 20s, 30s, 40s, 50s, 60s, 70s | 10s, 20s, 30s, 40s, 50s, | 20s, 30s, 40s, 50s, 60s, 70s | 10s, 20s, 30s, 40s, 60s, 70s | 20s, 30s, 40s, 50s, 60s, 70s | 10s, 20s, 30s, 40s, 50s, 60s, 70s |
|  | female (8,285) | 20s, 30s, 40s, 50s, 60s, 70s | 10s, 20s, 30s, 40s, 50s, 60s, 70s | 10s, 20s, 30s, 40s, 50s | 30s, 40s, 50s, 60s, 70s | 10s, 20s, 30s, 40s, 50s | 40s, 70s | 10s, 20s, 30s, 40s, 50s, 60s, 70s |

Table S4: Sleep durations (TST) by the American National Sleep Foundation (NSF) age groups

| Age group | Sample number | Recommended rage | Within range | Sleeping less | Sleeping more |
| --- | --- | --- | --- | --- | --- |
|  |  | by NSF | [%] | [%] | [%] |
| 1–2 years | 12 | 11–14 hours | - | - | - |
| 3–5 years | 21 | 10–13 hours | - | - | - |
| 6–13 years | 530 | 9–11 hours | 23.5% | 74.7% | 1.8% |
| 14–17 years | 741 | 8–10 hours | 25.0% | 67.7% | 7.4% |
| 18–25 years | 963 | 7–9 hours | 38.0% | 42.6% | 19.4% |
| 26–40 years | 3,962 | 7–9 hours | 38.9% | 49.6% | 11.4% |
| 41–65 years | 20,446 | 7–9 hours | 32.4% | 58.4% | 9.2% |
| >65 years | 44,036 | 7–8 hours | 20.1% | 46.3% | 33.6% |

Values were not calculated if sample number was less than 100 in each cell.

Table S5: Mean and standard error of mean for sleep parameters stratified by the American National Sleep Foundation age groups

| Age group | Strata by gender | TIB | In-bed time | Get-up time | TST | SE | SL | WASO |
| --- | --- | --- | --- | --- | --- | --- | --- | --- |
|  | (sample number) | [min] | [hh:mm] | [hh:mm] | [min] | [%] | [min] | [min] |
| 1–2 years | total (12) | - | - | - | - | - | - | - |
|  | male (7) | - | - | - | - | - | - | - |
|  | female (5) | - | - | - | - | - | - | - |
| 3–5 years | total (21) | - | - | - | - | - | - | - |
|  | male (10) | - | - | - | - | - | - | - |
|  | female (10) | - | - | - | - | - | - | - |
| 6–13 years | total (530) | 510.0 ± 4.5 | 22:23 ± 0:04 | 6:53 ± 0:04 | 471.8 ± 4.7 | 94.2 ± 0.4 | 12.2 ± 0.9 | 27.5 ± 1.7 |
|  | male (259) | 505.8 ± 6.4 | 22:20 ± 0:06 | 6:45 ± 0:06 | 464.5 ± 6.4 | 93.9 ± 0.5 | 13.3 ± 1.5 | 29.8 ± 2.5 |
|  | female (268) | 514.1 ± 6.3 | 22:27 ± 0:05 | 7:01 ± 0:05 | 478.7 ± 7.0 | 94.4 ± 0.6 | 11.2 ± 1.0 | 25.4 ± 2.5 |
| 14–17 years | total (741) | 474.8 ± 4.5 | 23:23 ± 0:05 | 7:18 ± 0:05 | 431.1 ± 4.3 | 93.6 ± 0.3 | 14.2 ± 0.7 | 29.4 ± 1.5 |
|  | male (401) | 482.3 ± 6.2 | 23:25 ± 0:06 | 7:27 ± 0:06 | 439.1 ± 5.9 | 93.9 ± 0.4 | 14.3 ± 0.9 | 28.9 ± 1.9 |
|  | female (338) | 466.2 ± 6.4 | 23:20 ± 0:07 | 7:06 ± 0:07 | 421.7 ± 6.2 | 93.3 ± 0.5 | 13.9 ± 1.1 | 30.1 ± 2.5 |
| 18–25 years | total (963) | 484.1 ± 4.8 | 23:40 ± 0:05 | 7:44 ± 0:06 | 441.8 ± 4.4 | 94.4 ± 0.3 | 15.4 ± 0.7 | 27.0 ± 1.4 |
|  | male (457) | 484.5 ± 7.3 | 23:50 ± 0:08 | 7:54 ± 0:09 | 442.2 ± 6.7 | 94.5 ± 0.4 | 16.0 ± 1.3 | 26.6 ± 2.0 |
|  | female (502) | 482.4 ± 6.1 | 23:33 ± 0:07 | 7:35 ± 0:08 | 441.1 ± 5.8 | 94.5 ± 0.4 | 14.9 ± 0.8 | 26.5 ± 1.8 |
| 26–40 years | total (3,962) | 455.9 ± 2.0 | 23:23 ± 0:02 | 6:59 ± 0:02 | 412.4 ± 1.9 | 93.5 ± 0.1 | 15.3 ± 0.4 | 28.5 ± 0.7 |
|  | male (1,726) | 465.9 ± 3.2 | 23:20 ± 0:04 | 7:06 ± 0:04 | 418.7 ± 3.0 | 93.3 ± 0.2 | 17.3 ± 0.7 | 30.3 ± 1.0 |
|  | female (2,221) | 447.9 ± 2.5 | 23:25 ± 0:03 | 6:53 ± 0:03 | 407.3 ± 2.5 | 93.8 ± 0.2 | 13.8 ± 0.4 | 27.1 ± 0.9 |
| 41–65 years | total (20,466) | 448.1 ± 0.9 | 22:50 ± 0:01 | 6:18 ± 0:01 | 396.8 ± 0.8 | 91.8 ± 0.1 | 15.6 ± 0.2 | 36.1 ± 0.3 |
|  | male (9,748) | 466.9 ± 1.4 | 22:33 ± 0:02 | 6:20 ± 0:01 | 414.9 ± 1.3 | 92.3 ± 0.1 | 16.5 ± 0.2 | 35.6 ± 0.5 |
|  | female (10,653) | 430.9 ± 1.1 | 23:05 ± 0:01 | 6:16 ± 0:01 | 380.3 ± 1.1 | 91.3 ± 0.1 | 14.6 ± 0.2 | 36.6 ± 0.5 |
| >65 years | total (44,036) | 491.5 ± 0.8 | 21:51 ± 0:01 | 6:03 ± 0:01 | 431.3 ± 0.7 | 90.5 ± 0.1 | 14.4 ± 0.1 | 46.0 ± 0.3 |
|  | male (18,498) | 506.4 ± 1.2 | 21:37 ± 0:01 | 6:03 ± 0:01 | 450.9 ± 1.1 | 91.8 ± 0.1 | 14.5 ± 0.2 | 41.0 ± 0.4 |
|  | female (25,454) | 480.7 ± 1.0 | 22:02 ± 0:01 | 6:02 ± 0:01 | 417.0 ± 0.9 | 89.4 ± 0.1 | 14.3 ± 0.2 | 49.6 ± 0.4 |

Values were not calculated if sample number was less than 100 in each cell. Values are represented as mean ± SEM.

**References**

1 Li, L. & Nakamura, T. An epidemiological sleep study based on a large-scale physical activity database. *The 2019 IEEE 1st Global Conference on Life Sciences and Technologies (LifeTech2019)*, 292-293 (2019).

2 Cole, R. J., Kripke, D. F., Gruen, W., Mullaney, D. J. & Gillin, J. C. Automatic sleep/wake identification from wrist activity. *Sleep* **15**, 461-469, doi:10.1093/sleep/15.5.461 (1992).

3 Karantonis, D. M., Narayanan, M. R., Mathie, M., Lovell, N. H. & Celler, B. G. Implementation of a real-time human movement classifier using a triaxial accelerometer for ambulatory monitoring. *Ieee T Inf Technol B* **10**, 156-167, doi:10.1109/titb.2005.856864 (2006).

4 Lugade, V., Fortune, E., Morrow, M. & Kaufman, K. Validity of using tri-axial accelerometers to measure human movement - Part I: Posture and movement detection. *Med Eng Phys* **36**, 169-176, doi:10.1016/j.medengphy.2013.06.005 (2014).

5 Ancoli-Israel, S. *et al.* The SBSM Guide to Actigraphy Monitoring: Clinical and Research Applications. *Behav Sleep Med* **13 Suppl 1**, S4-S38, doi:10.1080/15402002.2015.1046356 (2015).

6 Cohen, L. *Time-frequency analysis*. (Prentice Hall PTR, 1995).

7 Bishop, C. M. *Pattern recognition and machine learning*. (Springer, 2006).

8 Cortes, C. & Vapnik, V. Support-Vector Networks. *Machine Learning* **20**, 273-297, doi:Doi 10.1007/Bf00994018 (1995).

9 Cover, T. M. & Hart, P. E. Nearest Neighbor Pattern Classification. *IEEE Transactions on Information Theory* **13**, 21-27, doi:Doi 10.1109/Tit.1967.1053964 (1967).

10 Arthur, D. & Vassilvitskii, S. K-Means++: The advantages of careful seeding. *the 8th annual ACM-SIAM symposium on Discrete algorithms (2007)* **8**, 1027-1035, doi:10.1145/1283383.1283494 (2007).

11 Goutte, C. & Gaussier, E. *A probabilistic interpretation of precision, recall and F-score, with implication for evaluation*. Vol. 3408 (2005).

12 Rudin, L. I., Osher, S. & Fatemi, E. Nonlinear Total Variation Based Noise Removal Algorithms. *Physica D* **60**, 259-268, doi:Doi 10.1016/0167-2789(92)90242-F (1992).

13 Selesnick, I. W., Graber, H. L., Pfeil, D. S. & Barbour, R. L. Simultaneous Low-Pass Filtering and Total Variation Denoising. *IEEE Transactions on Signal Processing* **62**, 1109-1124, doi:10.1109/TSP.2014.2298836 (2014).

14 Condat, L. A Direct Algorithm for 1-D Total Variation Denoising. *IEEE Signal Processing Letters* **20**, 1054-1057, doi:10.1109/Lsp.2013.2278339 (2013).

15 Selesnick, I. W., Arnold, S. & Dantham, V. R. Polynomial Smoothing of Time Series With Additive Step Discontinuities. *IEEE Transactions on Signal Processing* **60**, 6305-6318, doi:10.1109/Tsp.2012.2214219 (2012).

16 Chambolle, A. An algorithm for total variation minimization and applications. *Journal of Mathematical Imaging and Vision* **20**, 89-97, doi:10.1023/B:JMIV.0000011325.36760.1e (2004).

17 Fekedulegn, D. *et al.* Actigraphy-Based Assessment of Sleep Parameters. *Ann Work Expo Health* **64**, 350-367, doi:10.1093/annweh/wxaa007 (2020).

18 Webster, J. B., Kripke, D. F., Messin, S., Mullaney, D. J. & Wyborney, G. An activity-based sleep monitor system for ambulatory use. *Sleep* **5**, 389-399, doi:10.1093/sleep/5.4.389 (1982).

19 Conley, S. *et al.* Agreement between actigraphic and polysomnographic measures of sleep in adults with and without chronic conditions: A systematic review and meta-analysis. *Sleep Medicine Reviews* **46**, 151-160, doi:10.1016/j.smrv.2019.05.001 (2019).

20 de Souza, L. *et al.* Further validation of actigraphy for sleep studies. *Sleep* **26**, 81-85, doi:DOI 10.1093/sleep/26.1.81 (2003).

21 Martin Bland, J. & Altman, D. Statistical Methods for Assessing Agreement between Two Methods of Clinical Measurement. *The Lancet* **327**, 307-310, doi:10.1016/s0140-6736(86)90837-8 (1986).
